# Widespread employment of conserved *C*. *elegans* homeobox genes in neuronal identity specification

**DOI:** 10.1371/journal.pgen.1010372

**Published:** 2022-09-30

**Authors:** Molly B. Reilly, Tessa Tekieli, Cyril Cros, G. Robert Aguilar, James Lao, Itai Antoine Toker, Berta Vidal, Eduardo Leyva-Díaz, Abhishek Bhattacharya, Steven J. Cook, Jayson J. Smith, Ismar Kovacevic, Burcu Gulez, Robert W. Fernandez, Elisabeth F. Bradford, Yasmin H. Ramadan, Paschalis Kratsios, Zhirong Bao, Oliver Hobert

**Affiliations:** 1 Department of Biological Sciences, Columbia University, Howard Hughes Medical Institute, New York, New York, United States of America; 2 Department of Neurobiology, University of Chicago, Chicago, Illinois, United States of America; 3 Developmental Biology Program, Sloan Kettering Institute, New York, New York, United States of America; University of California San Diego, UNITED STATES

## Abstract

Homeobox genes are prominent regulators of neuronal identity, but the extent to which their function has been probed in animal nervous systems remains limited. In the nematode *Caenorhabditis elegans*, each individual neuron class is defined by the expression of unique combinations of homeobox genes, prompting the question of whether each neuron class indeed requires a homeobox gene for its proper identity specification. We present here progress in addressing this question by extending previous mutant analysis of homeobox gene family members and describing multiple examples of homeobox gene function in different parts of the *C*. *elegans* nervous system. To probe homeobox function, we make use of a number of reporter gene tools, including a novel multicolor reporter transgene, NeuroPAL, which permits simultaneous monitoring of the execution of multiple differentiation programs throughout the entire nervous system. Using these tools, we add to the previous characterization of homeobox gene function by identifying neuronal differentiation defects for 14 homeobox genes in 24 distinct neuron classes that are mostly unrelated by location, function and lineage history. 12 of these 24 neuron classes had no homeobox gene function ascribed to them before, while in the other 12 neuron classes, we extend the combinatorial code of transcription factors required for specifying terminal differentiation programs. Furthermore, we demonstrate that in a particular lineage, homeotic identity transformations occur upon loss of a homeobox gene and we show that these transformations are the result of changes in homeobox codes. Combining the present with past analyses, 113 of the 118 neuron classes of *C*. *elegans* are now known to require a homeobox gene for proper execution of terminal differentiation programs. Such broad deployment indicates that homeobox function in neuronal identity specification may be an ancestral feature of animal nervous systems.

## Author summary

We address here how neuron types acquire their specific molecular identity features. We focus on a specific family of transcription factors, encoded by homeobox genes and analyze the function of more than a dozen homeobox gene family members throughout the entire nervous system of the nematode *C*. *elegans*, which is composed of 118 different neuron classes. We discover homeobox gene functions in 24 distinct neuron classes that are mostly unrelated by location, function and lineage history. Together with previous studies, this work demonstrates the wide-spread employment of this transcription factor family in neuronal identity specification, suggesting that these factors may have been recruited to neuronal cell differentiation early in evolution.

## Introduction

Nervous systems are composed of diverse sets of neuron types, each characterized by the expression of specific gene batteries that define the structural and functional features of that mature neuron type. A fundamental question in developmental neurobiology is whether there are common organizational principles for how individual neuron types acquire their unique identities. One approach to uncover such common principles is to comprehensively analyze neuronal differentiation programs throughout a given nervous system and determine whether a specific set of rules or features recurs in the specification of different neuron types in different parts of the organism. We have engaged in such holistic analysis in the nematode *C*. *elegans*, which contains a nervous system with substantial cellular diversity but limited overall number: 302 neurons in the hermaphrodite which fall into 118 classes [1]. In our search for common principles in *C*. *elegans* neuron type specification, several themes have emerged: (1) the direct, coordinated control of neuron type specific gene batteries by so-called “terminal selectors” [2–4]; (2) the overrepresentation of homeodomain transcription factors as terminal selectors [5,6] and (3) the codification of individual neuron identities by distinct combinations of homeodomain proteins, unique for each individual neuron type [5–7].

Since the DNA binding site of a single transcription factor does not encode enough specificity to select downstream targets genes in vast genome sequence space, transcription factors usually operate in combination with other transcription factors [8–10]. In the context of homeodomain proteins in the *C*. *elegans* nervous system, combinatorial functions of co-expressed homeodomain proteins have usually been inferred genetically through removal of co-expressed homeobox genes, either in isolation or in combination, resulting in neuronal differentiation defects. In some cases, the biochemical basis for such combinatorial activity has been deduced: For example, in the case of the gentle touch receptor neurons, the POU and LIM homeodomain proteins UNC-86 and MEC-3 bind cooperatively to target gene promoters to determine the fully differentiated state of these neurons [11]; similarly, the Prd and LIM homeodomain proteins CEH-10 and TTX-3, whose expression uniquely overlaps in the cholinergic AIY interneurons, show cooperative binding to *cis-*regulatory elements of members of the gene battery that define the terminally differentiated state of the AIY neurons [12]. In other cases, homeodomain proteins appear to bind to target gene promoters independently of one another, but their joint presence is required for target gene expression [13,14].

In this paper, we set out to further probe the extent to which homeobox genes, and combinations thereof, are involved in neuronal identity specification. First, we further refine our atlas of homeodomain protein expression throughout the nervous system and, second, we define the impact of loss-of-function alleles of 12 homeobox genes on neuronal identity specification throughout the entire nervous system. One pillar of this mutant analysis is the recently described NeuroPAL transgene, which expresses more than 40 distinct neuronal identity markers throughout the entire *C*. *elegans* nervous system [15]. Together with other available molecular markers we identify 14 homeobox genes involved in the specification of 24 different neuron classes (of the total of 118 *C*. *elegans* hermaphrodite neuron classes), 12 of which had no previously known regulator assigned to them. This mutant analysis therefore expands our understanding of homeobox gene function in neuronal identity control, arguing that homeobox genes play a central and perhaps ancestral role in neuronal identity specification.

## Results

### Reporter alleles refine some homeodomain protein expression profiles

Precise knowledge of the expression pattern of a gene provides a useful guide for mutant analysis. In our previous, genome-wide analysis of homeodomain protein expression, we made use of both CRISPR/Cas9-engineered reporter alleles, as well as fosmid-based reporters to assess expression patterns [6]. Fosmids are generally 30–50 kb genomic fragments, usually containing several genes up/downstream of a gene of interest and can be expected to include all *cis*-regulatory information of a tagged locus. Indeed, the expression pattern of many fosmid reporters is successfully recapitulated by CRISPR/Cas9 genome-engineered reporter alleles [7,16–18]. A recently published nervous system wide scRNA transcriptome atlas, called CeNGEN [19] is also largely congruent with an atlas of homeodomain expression profiles that was based on either fosmid-based reporters or CRISPR/Cas9-engineered reporter alleles [6]. We nevertheless set out to compare the expression of 18 newly available CRISPR/Cas9-engineered reporter alleles, generated either by us or obtained from the Du lab [20], with previously described fosmid-based reporter patterns and the scRNA CeNGEN atlas (Table 1). Like the fosmid reporters, these reporter alleles fuse a reporter directly to the N- or C-terminus of the encoded homeodomain protein, thereby allowing the direct monitoring of protein expression.

**Table 1:** Comparing expression patterns of CRISPR/Cas9-engineered reporter alleles with fosmid-based reporter transgenes.

Homeobox gene	Reporter allele ^1^	CRISPR/Cas9-engineered reporter allele		Only observed with fosmid reporter ^2^, not with reporter allele
		also observed with fosmid reporter ^2^	not observed with fosmid	
*vab-3*	*devKi190*	ASK, BAG, OLQ, CEP, RIF, RIV**,** AS11, VA11, VD12	ADA, OLL, URA, URB, URY	PVM*, SMB*, ALM, PLM, AVD, DBs, VBs, SAA
*lim-7*	*devKi125*	RIA, URY, OLL, PVN, PHC, AVB, AIB, AIM, BAG, M1, M5	ALA, MI, AWC, I3, I4, BAG	AFD*, PVC*, RME
*ceh-27*	*syb2714*	I6, RIM, RIP, RME, RMF	AVL	none
*ceh-43*	*syb5073*	ADE, AIZ, ASJ, BDU, CAN, CEP, IL1, PDE, PVQ, SDQ	AIN	URB
*ceh-45*	*devKi191*	I1, MI	RIB	none
*ceh-30*	*syb4678*	SDQ	none	ubiquitous
*ceh-31*	*devKi250*	URA, URB, AVB, SDQ, PVR	none	none
*ceh-16*	*syb2880*	AIZ, RIF, RIG	none	none
*ceh-36*	*syb2934*	ASE, AWC, ASI, AWA	none	none
*ceh-12*	*devKi186*	VB	none	none
*unc-30*	*hzhCR1*	ASG, AVJ, DD, PVP, VD	none	none
*ceh-17*	*devKi180*	ALA, SIA	none	none
*ceh-5*	*devKi103*	embryo only	none	none
*ceh-51*	*devKi72*	embryo only	none	none
*ceh-82*	*devKi181*	none	none	ubiquitous
*ceh-28*	*devKi192*	M4	none	none
*ceh-2*	*devKi17*	I3, M3	none	NSM (dim)
*ceh-10*	*devKi101*	AIY, CAN, RID, DVC ^3^	N/A ^3^	N/A ^3^

Underlined neuron classed names indicate scRNA transcript found in this neuron in CeNGEN scRNA dataset [19]. When marked with *, transcript levels were very low, compared to other neurons that express the gene.

^1^ Generated by CRISPR/Cas9-based genome engineering. *ot* alleles were generated in our lab, *syb* alleles by Sunybiotech, *dev* and *hzh* alleles were kindly provided by Zhuo Du.

^2^ As described in [6]

^3^ the previously reported expression pattern [6] was not based on a fosmid but on a personal communication about another reporter allele.

**Fig 1:**
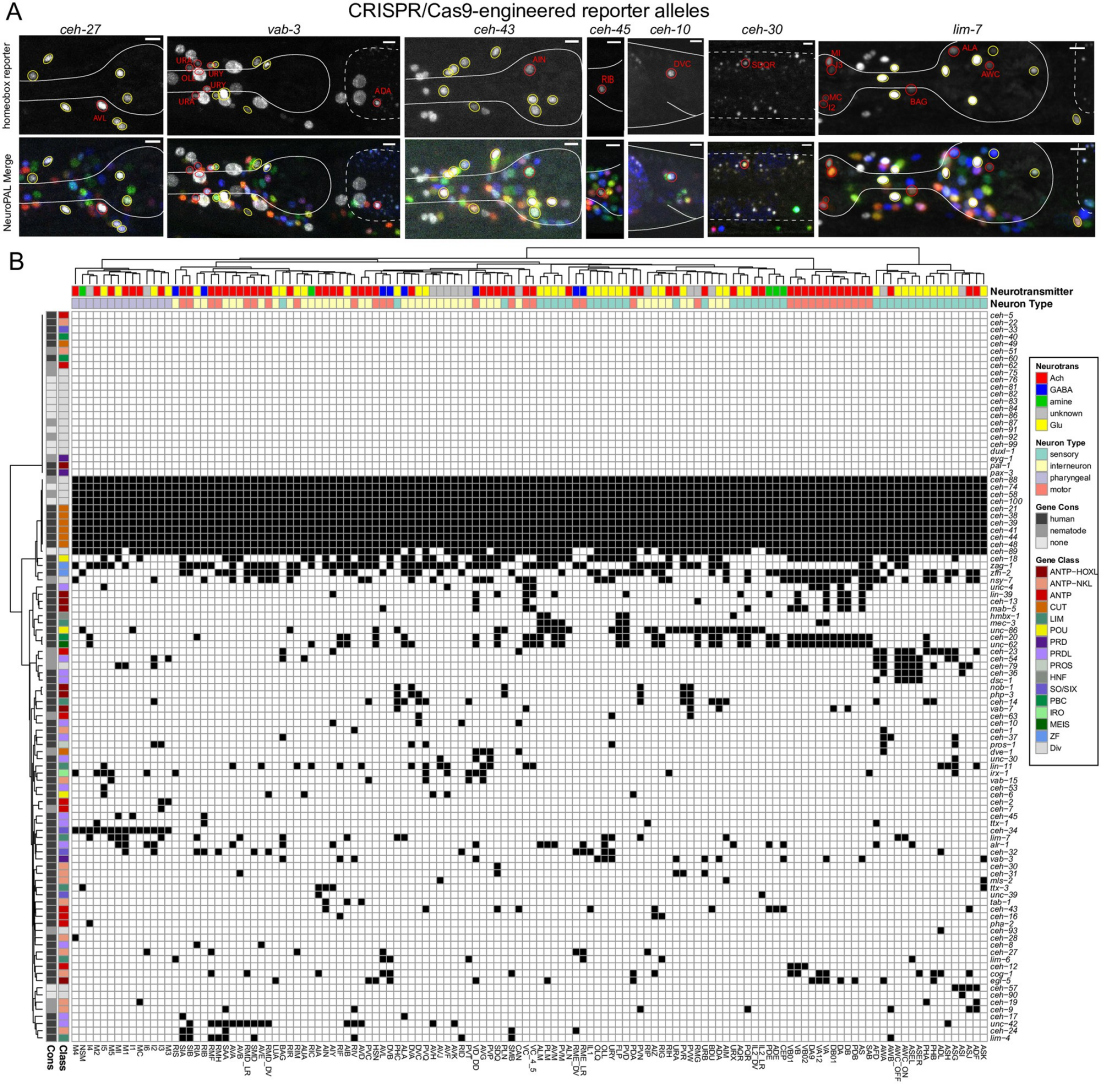
Updated expression of the homeobox gene family with reporter alleles. **Fig 1A:** Representative images of homeobox reporter alleles, generated by CRISPR/Cas9 genome engineering (see strain list in S5 Table) with different expression than previously reported fosmid-based reporter transgenes. Neuron classes showing expression not previously noted were identified by overlap with the NeuroPAL landmark strain, and are outlined and labeled in red. Neuron types in agreement with previous reporter studies are outlined in yellow. Head structures including the pharynx were outlined in white for visualization. Autofluorescence common to gut tissue is outlined with a white dashed line. An n of 10 worms were analyzed for each reporter strain. Scale in bottom or top right of the figure represents 5 μm. See also S1 Fig for more information on *ceh-30* and *ceh-31*. **Fig 1B:** Summary of expression of all homeobox genes across the *C*. *elegans* nervous system, taking into account new expression patterns from panel A and all previously published data [6]. Black boxes indicate that a homeodomain transcription factor is expressed in that given neuron type and white boxes indicate that a homeodomain transcription factor is not expressed in that given neuron type. Neuron types along the x axis are clustered by transcriptomic similarity using the Jaccard index (see methods) and homeobox genes along the y axis are clustered similarly by their similar expression profiles in shared neuron types. See S3 Fig for numerical representation of homeoboxes per neuron.

We observed expression patterns with the reporter alleles that are largely congruent between the fosmid-based expression profiles, but also observed some differences (Fig 1 **and** Table 1). Among the homeobox genes with the greatest difference in gene expression profiles are *vab-3*, the *C*. *elegans* Eyeless/PAX6 ortholog and *ceh-30*, one of the two *C*. *elegans* BarH1 homologs. In retrospect, the difference between the sites of expression of these fosmid reporters and the reporter alleles is not surprising. First, the previously used *vab-3* fosmid reporter stood out for its weakness and variability in expression. Second, the fosmid-based reporter for *ceh-30* covered all intergenic, non-coding regions, but it did not over all intergenic region of the neighboring paralogue *ceh-31* with whom *ceh-30* may share *cis-*regulatory control elements (Figs 1 **and** S1).

In two other cases, we observed that the reporter allele added expression in one additional neuron class (Fig 1 **and** Table 1). For example, a *ceh-45* reporter allele is expressed in I1 and MI, as previously reported with a fosmid-reporter [6], but additional, albeit weaker expression is observed in the RIB interneurons with the reporter allele. In all these cases, the additional expression was supported by scRNA CeNGEN data [19], but the expression level was notably lower in the respective neuron class. In these cases, the fosmid reporter may have lacked *cis*-regulatory element(s) or may have been too weakly expressed.

In one case, the EMX homolog *ceh-2*, we recapitulated previously reported I2 and M3 expression, but failed to observe CEH-2 protein expression in the NSM neuron, which had shown expression with the fosmid reporter [6]. Since this neuron class also shows scRNA transcripts of the *ceh-2* homeobox gene [19], it is conceivable that multicopy overexpression of the fosmid reporter may have titrated out a rate-limiting posttranscriptional regulatory event.

Altogether, the revised expression patterns of homeodomain proteins further refine the nature of combinatorial homeodomain expression codes that uniquely define each neuron class (Fig 1B **and** S1 and S2 Tables). *In toto*, 79 of the 102 *C*. *elegans* homeobox genes are expressed in the nervous system, 69 of which on a neuron type-specific manner, with neuron-type specific combinatorial codes [6,21] (this paper). We display this combinatorial coding data in a manner distinct from our previous study [6]. We first clustered neuronal cell types by similarity of scRNA-generated gene expression profiles (see Methods; S2 Fig) and then mapped homeodomain expression patterns onto this matrix. This representation provides a visually tractable way to discern whether transcriptionally similar neurons tend to express the same homeodomain protein(s) (Fig 1B **and** S1 **and** S2 Tables). There are indeed several instances of such co-clustering. For example, the set of neurons that co-express the homeodomain protein CEH-34 and the set of neurons that co-express the homeodomain protein UNC-42 share more molecular similarities among themselves than with other neuron classes (Fig 1B). This is particularly notable because both the UNC-42(+) and CEH-34(+) neurons are synaptically more interconnected than expected by chance [7, 17].

### Mutant analysis of homeobox genes

Guided by homeodomain protein expression patterns, we sought to further implicate them in the process of terminal neuron differentiation using homeobox mutant strains. It is important to emphasize that our homeodomain protein expression profiles are entirely focused on those proteins that are continuously expressed throughout the life of a neuron and are therefore candidates to not only initiate but also maintain the differentiated state of a neuron, the original criterion for being a terminal selector of neuronal identity [2,4]. Our mutant analysis covered neurons that fall into three categories (Table 2): (1) neurons for which no transcriptional identity regulator (terminal selector) had been described before; (2) neurons for which only a non-homeobox identity regulator had been identified; (3) neurons for which no unique functional combination of homeobox genes has been identified. The neurons that we covered in this analysis are functionally diverse, many have different lineage histories and are located in distinct parts of the *C*. *elegans* nervous system (Table 2).

**Table 2:** Summary of newly identified homeobox regulators of neuronal differentiation.

Neuron class	type	location	lineage history	Previously identified identity regulator for this neuron class	Newly described homeobox gene required for identity (this paper)
no identity regulator known before					
AVJ	peptidergic interneuron	lateral ganglion	ABalap(a/p)pppa	none	*lin-11/LHX1*
					*unc-30/PITX*
					*mls-2/HMX*
ADA	glutamatergic interneuron	anterior deirid lineage	ABp(l/r)apaaaapp	none	*ceh-14/LHX3*
					*unc-86/BRN3*
					*unc-62/MEIS*
RMG	peptidergic interneuron	anterior deirid lineage	ABp(l/r)apaaapp	none	*unc-86/BRN3*
					*ceh-13/HOX*
RIC	octopaminergic interneuron	lateral ganglion	ABp(l/r)ppaaaapp	none	*unc-62/MEIS*
RIR	cholinergic interneuron	ventral ganglion	ABprpapppaa	none	*unc-86/POU*
RIP	peptidergic interneuron	anterior ganglion	AB alpapaaaa, AB arappaaaa	none	*ttx-1/OTX*
RIB	GABAergic interneuron	lateral ganglion	AB p(l/r)paappap	none	*ttx-1/OTX*
AIN	cholinergic interneuron	lateral ganglion	ABalaaaalal ABalaapaaar	none	*tab-1/BSX*
OLQ	glutamatergic sensory	anterior ganglion	ABalap(a/p)papaa ABp(l/r)paaappaa	none	*vab-3/PAX6*
IL1	glutamatergic sensory	anterior ganglion	ABalapappaaa ABalappppaaa ABalapaappaa ABalaappppaa ABalppapppaa ABarapppppaa	none ^1^	*ceh-32/SIX3/6*
PVW	peptidergic interneuron	lumbar ganglion	T(L/R).ppa	none	*ceh-14/LHX3*
LUA	glutamatergic interneuron	lumbar ganglion	ABp(l/r)pppaapap	none	*egl-5/HOX*
only non-homeobox regulator known before					
AWA	peptidergic sensory	lateral ganglion	ABp(l/r)aapapaa	*odr-7* (nuclear receptor)	*egl-5/HOX*
RME	GABAergic motor neuron	nerve ring	ABalapppaap ABalaaaar(l/r)p ABplpappaaa	*nhr-67/TLX* (nuclear receptor)	*ceh-32/SIX3*
PDA	cholinergic motor neuron		ABprpppaaaa	*unc-3/COE* (Zn finger)	*egl-5/HOX*
complements incomplete combination of previously described homeobox genes					
AIA	cholinergic interneuron	ventral ganglion	ABp(l/r)ppaappa	*ttx-3/LHX2*	*unc-39/SIX4*
AIM	glutamatergic interneuron	ventral ganglion	ABp(l/r)paapppa	*unc-86/BRN3*	*mls-2/HMX* *ceh-14/LHX3*
AIZ	glutamatergic interneuron	anterior deirid lineage	AB p(l/r)apaaapav	*unc-86/BRN3*	*ceh-43/DLX*
AQR	glutamatergic sensory neuron	head	QR.ap	*unc-86/BRN3* *lin-39/HOX* *egl-13/SoxD*	*unc-62/MEIS* *ceh-20/PBX*
FLP	glutamatergic sensory neuron	anterior deirid lineage	ABp(l/r)apaaapad	*unc-86/BRN3* *mec-3/LHX*	*unc-62/MEIS* *ceh-20/PBX*
AVD	glutamatergic interneuron	lateral ganglion	ABalaaapalr ABalaaapprl	*unc-42/PROP1* *unc-3/COE* *cfi-1/ARID*	*tab-1/BSX*
OLL	glutamatergic sensory	anterior ganglion	ABalppppapaa ABpraaapapaa	*vab-3/PAX6*	*ceh-32/SIX3*
URY	glutamatergic sensory	anterior ganglion	ABalap(a/p)papp ABp(l/r)paaappp	*vab-3/PAX6*	*ceh-32/SIX3*
URB	cholinergic interneuron	anterior ganglion	AB plaapaapa AB praapaapa	*unc-86/BRN3*	*vab-3/PAX6*
URA	cholinergic interneuron	anterior ganglion	AB plaaaaaaa, AB arpapaaaa, AB plpaaapaa, AB prpaaapaa	*unc-86/BRN3*	*vab-3/PAX6*

^1^ we had previously assumed *vab-3* to be a candidate terminal selector for the IL1 neurons [48]; however, more recent expression pattern analysis indicates that *vab-3* is not continuously expressed in IL1 [6]. Hence, the reported IL1 differentiation defects of *vab-3* mutants are likely a reflection of earlier function of *vab-3* in the lineage.

### The SIX-type homeobox gene *unc-39* affects differentiation of the AIA interneuron class

**Fig 2:**
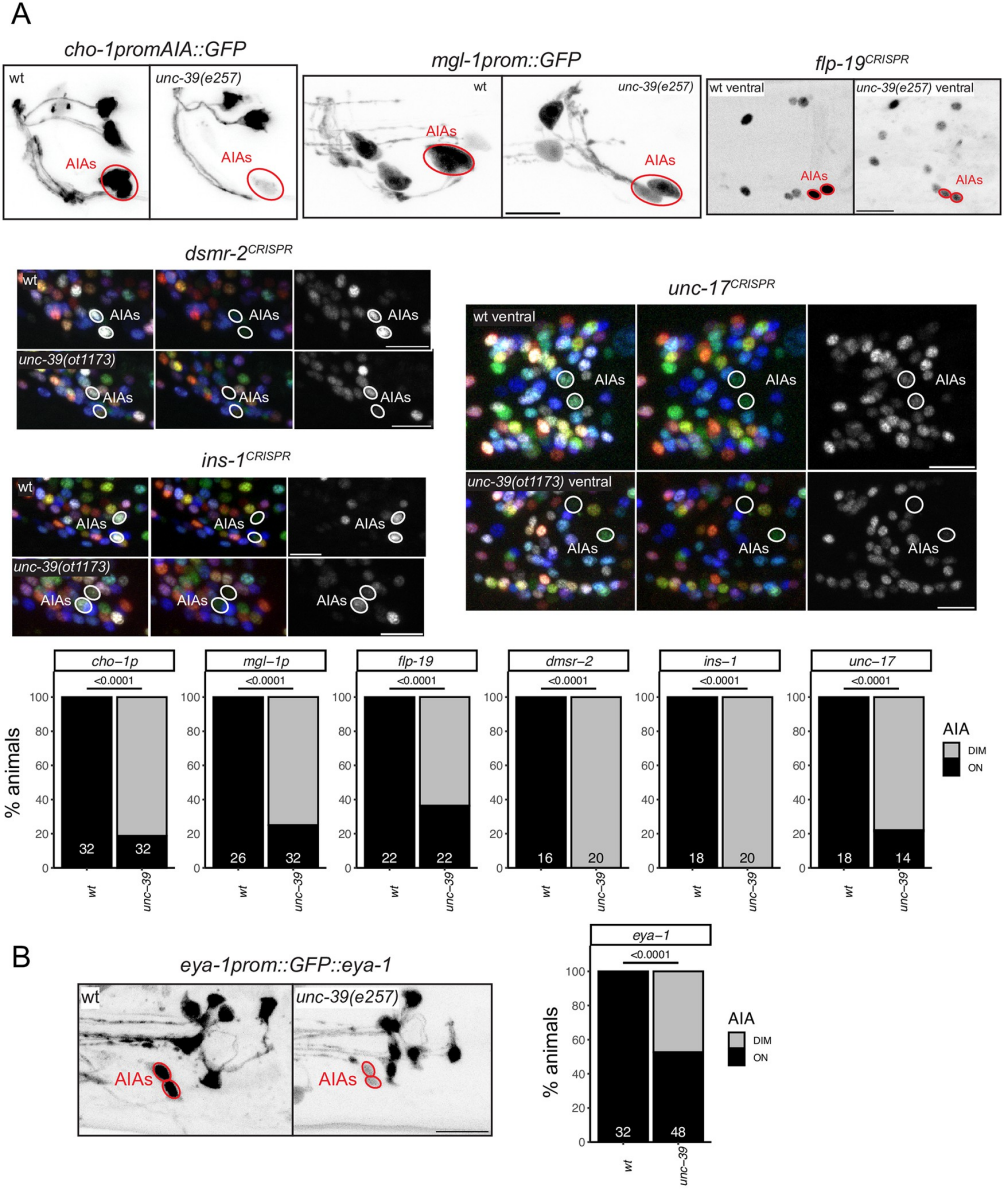
*unc-39* controls differentiation of the AIA interneuron class. *unc-39*^R203Q^ mutant animals (either canonical *e257* allele or CRISPR/Cas9 genome engineered *ot1173* allele with identical nucleotide change) were analyzed. **Fig 2A:** *unc-39* affects the cholinergic identity of the AIA interneuron class (*unc-17* reporter allele *syb4491* and a *cho-1* promoter fragment which is part of the *otIs653* array), and other AIA terminal identity markers: reporter alleles *dmsr-2(syb4514)*, *ins-1(syb5452)* and *flp-19(syb3278)*, and a *mgl-1* promoter fragment *otIs327*. We did not quantify changes in AIA in the NeuroPAL color code, because it is variable in wild type. Representative images of wild type and mutant worms are shown with 10 μm scale bars. Graphs compare expression in wild type and mutant worms with the number of neurons examined listed at the bottom of the bar. P-values were calculated by Fisher’s exact test. **Fig 2B:** *unc-39* affects the expression of the tagged *eya-1* locus (*nIs352* transgene) in AIA.

We have previously reported that the LIM homeobox gene *ttx-3* is required for the proper differentiation of the cholinergic AIY and AIA interneurons [14]. However, unlike in the AIY interneuron class, where TTX-3 partners with the Chx10 homolog CEH-10 [12], no homeodomain protein was shown to be a functional partner for TTX-3 in the AIA neurons. The Six4/5 homeodomain protein UNC-39 is continuously expressed in the AIA interneurons from their birth, throughout the animal’s life [6], making it a candidate TTX-3 cofactor. The only other site of UNC-39 expression is the lateral IL2 neuron subtype [6]. We found that *unc-39(e257)* mutant animals display strong defects in the expression of six out of six tested molecular markers of AIA identity, two neuropeptides (*ins-1* and *flp-19)*, the ACh vesicular transporter, *unc-17*, the choline reuptake transporter *cho-1*, a neuropeptide receptor, *dmsr-2* and a metabotropic glutamate receptor, *mgl-1* (Fig 2A), indicating that UNC-39 may indeed cooperate with TTX-3 to specify AIA identity.

The Eyes Absent protein is a transcriptional co-factor for many SIX domain-type homeodomain proteins across phylogeny [7,22–26]. Despite the expression of several SIX domain family members in many different parts of the *C*. *elegans* nervous system [6], a genomic fragment that contains the entire, *gfp-*tagged *eya-1* locus [27] is only expressed in pharyngeal neurons [7] and one single extra-pharyngeal neuron class, the AIA neuron class (Fig 2B). This pattern is supported by scRNA analysis [19]. *unc-39* is required for proper *eya-1* expression in the AIA neurons (Fig 2B). However, using two different markers (*unc-17*, *flp-19*), we observe no AIA differentiation defects in *eya-1* null mutants (S4 Fig).

### The LIM homeobox gene *ceh-14* specifies distinct neuron classes

**Fig 3:**
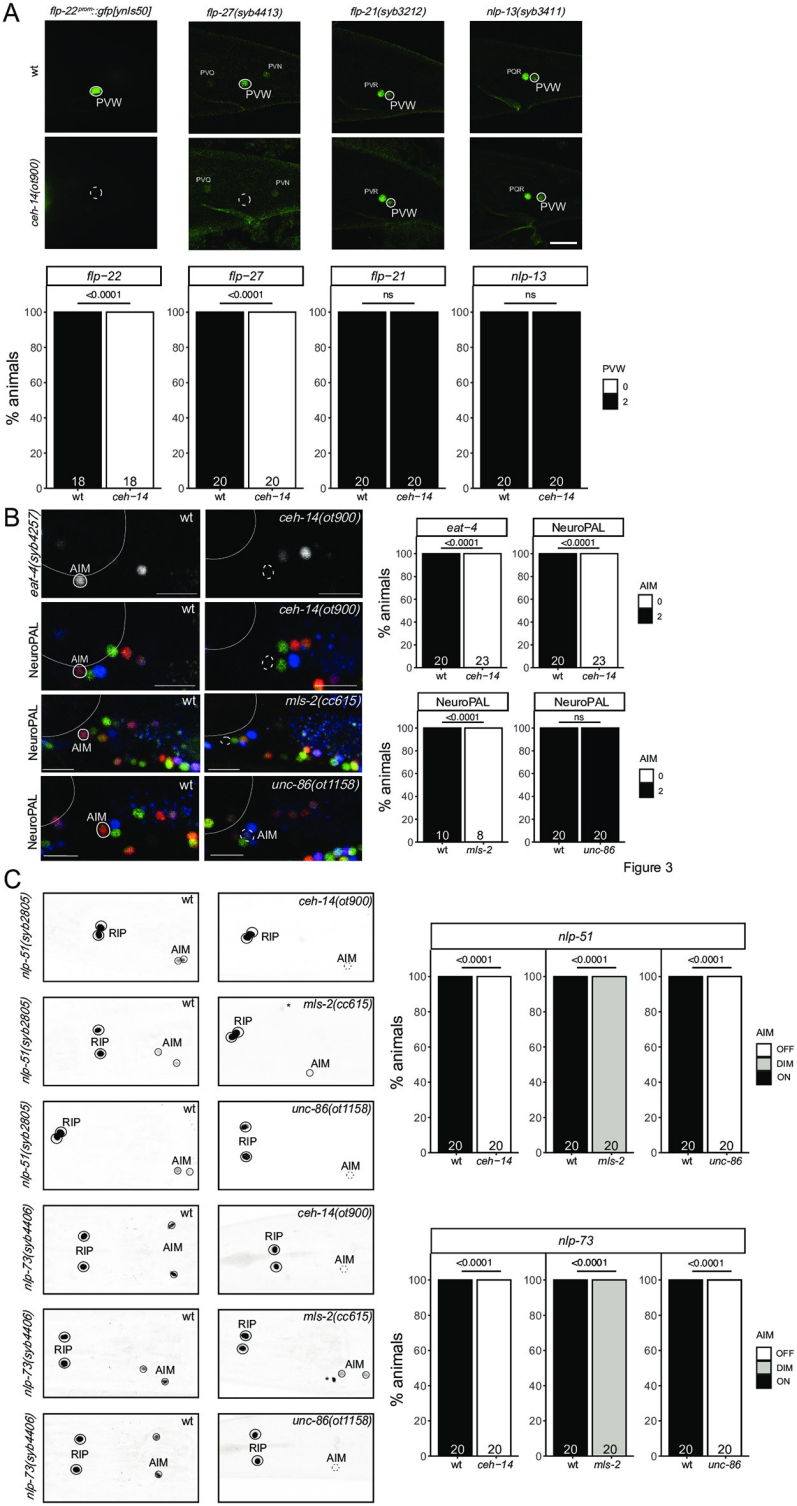
*ceh-14* affect differentiation of several neuron classes, in combination with different homeobox genes. **Fig 3A:** *ceh-14(ot900)* mutant animals show a loss of neuropeptide-encoding gene expression in PVW, including a promoter fusion reporter transgene for *flp-22* (*ynIs50*) and a *flp-27* CRISPR reporter (*syb4413*), while expression of the neuropeptide CRISPR reporters for *flp-21* (*syb3212*) and *nlp-13* (*syb3411*) is unaffected. Neuron of interest is outlined in solid white when expressing wildtype reporter colors, and dashed white when one or all colors are lost. Representative images of wild type and mutant worms are shown with 10 μm scale bar. Graphs compare expression in wild type and mutant worms with the number of animals examined listed at the bottom of the bar. P-values were calculated by Fisher’s exact test. **Fig 3B:** *ceh-14(ot900)* mutant animals show a loss of AIM marker expression, including an *eat-4* CRISPR reporter (*syb4257*) and NeuroPAL (*otIs669*) in AIM. Additionally, *unc-86(ot1158)* as well as *mls-2(cc615)* mutant animals show expression defects of NeuroPAL (*otIs669)* in AIM. Neuron of interest is outlined in solid white when expressing wildtype reporter colors, and dashed white when one or all colors are lost. Representative images of wild type and mutant worms are shown with 10 μm scale bars. Graphs compare expression in wild type and mutant worms with the number of animals examined listed at the bottom of the bar. P-values were calculated by Fisher’s exact test. **Fig 3C:** *ceh-14(ot900)* and *unc-86(ot1158)* mutant animals show a loss of neuropeptide-encoding gene expression in AIM using the CRISPR/Cas9-engineered reporter alleles *nlp-51(syb2805)* and *nlp-73(syb4406)*. *mls-2(cc615)* mutant animals diminish, but do not extinguish expression in of *nlp-51(syb2805)* and *nlp-73(syb4406)* in AIM. Expression in RIP is unaffected by *ceh-14(ot900)*, *unc-86(ot1158)*, and *mls-2(cc615)*. Neurons are outlined in solid black and a dashed black line represents loss of expression. Asterisks indicate ectopic expression of unidentified neurons. Graphs compare expression in wild type and mutant worms (on vs off or on vs dim) with the number of animals examined listed at the bottom of the bar. P-values were calculated by Fisher’s exact test.

The PVW neuron pair is a peptidergic interneuron class located in the lumbar ganglion in the tail of the animal, with unknown function and no known identity regulator. The PVW neuron pair continuously expresses the *C*. *elegans* homolog of the vertebrate LHX3/4 LIM homeobox gene *ceh-14* [6,28]. We assessed the peptidergic identity of PVW by analyzing the expression of four different neuropeptide-encoding genes that the CeNGEN scRNA atlas predicts to be expressed in PVW, *flp-21*, *flp-22*, *flp-27* and *nlp-13* [19]. To monitor *flp-22* expression, we used a previously available promoter fusion transgene [29]. For the other three neuropeptides, an SL2::gfp::H2B or T2A::3xNLS::gfp cassette was inserted at the C-terminus of the respective loci. All four reporters show expected expression in PVW (Fig 3A). In *ceh-14* null mutant animals, expression of two of the four neuropeptide-encoding genes (*flp-22* and *flp-27*) is lost (Fig 3B).

We also analyzed *ceh-14* function in a completely distinct set of interneurons in which CEH-14 protein is continuously expressed, the AIM neuron pair in the ventral ganglion of the head. We had previously shown that *ceh-14* affects neurotransmitter identity of AIM [30]. We confirmed these defects using an *eat-4/VGLUT* reporter allele, generated by CRISPR/Cas9 genome engineering and a previously unavailable molecular null allele of *ceh-14*, *ot900* (Fig 3B). We also analyzed expression of the NeuroPAL transgene, which expresses three markers for AIM identity, the ionotopic ACh receptor *acr-5*, the *mbr-1* transcription factor and *eat-4/VGLUT* [15]. The NeuroPAL transgene also contains a synthetic panneuronal marker, which permits assessment of the generation of a neuron. We find that in *ceh-14* null mutants, the neuron-type specific markers on the NeuroPAL transgene fail to be expressed in the AIM interneurons, while panneuronal markers are unaffected (Fig 3B). Lastly, using CRISPR/Cas9 genome engineering, we generated reporter alleles for two neuropeptide encoding genes, *nlp-51* and *nlp-73* for which the CeNGEN project detected expression in a small number of neurons, including AIM [19]. Both reporter alleles confirmed expression in AIM and showed loss of expression in AIM in *ceh-14* mutants (Fig 3C). Together, this data strongly indicates that *ceh-14* is a terminal selector of AIM identity.

Another homeobox gene continuously expressed in AIM is the HMX-type homeobox gene *mls-2*. *mls-2* expression overlaps with *ceh-14* exclusively in the AIM neurons. Previous work has shown that the neuropeptide-encoding gene, *flp-10*, fails to express in the AIM interneuron of *mls-2* mutants [31]. To determine whether *mls-2* has similar defects as *ceh-14* mutants, we assessed expression of the NeuroPAL transgene in the AIM neurons of *mls-2* mutants. We find that *mls-2* mutants display the same color loss of the NeuroPAL transgene as *ceh-14* mutants, suggesting that *ceh-14* and *mls-2* homeobox gene collaborate to instruct AIM identity (Fig 3B). Moreover, both AIM-expressed neuropeptide genes, *nlp-51* and *nlp-73* fail to be properly expressed in the AIM neuron of *mls-2* mutants (Fig 3C).

Lastly, the BRN3 ortholog *unc-86*, a POU homeobox gene, was previously also shown to affect glutamatergic identity of the AIM neurons, as assessed by loss of *eat-4/VGLUT* fosmid reporter expression [32]. Consistent with a role of *unc-86* in AIM differentiation, *unc-86* null mutants also display NeuroPAL color code defects (Fig 3B). In this case, however, the wild type color composition of NeuroPAL in AIM (dim *acr-5*::*mTagBFP*, bright *eat-4*::*mNeptune2*.*5*, dim *mbr-1*::*magenta*) changes from bright red to a more blue color, which corroborates loss of *eat-4/VGLUT* expression, but indicates a potential increase of expression of *acr-5* and *mbr-1* expression. The two neuropeptides *nlp-51* and *nlp-73* lose expression in AIM in *unc-86* mutants (Fig 3C). We conclude that *unc-86* also affects AIM differentiation, albeit in a manner subtly distinct from the effect of *ceh-14* and *mls-2*.

### *mls-2/HMX*, *unc-30/Pitx* and *lin-11/Lhx* specify the previously uncharacterized AVJ neuron class

**Fig 4:**
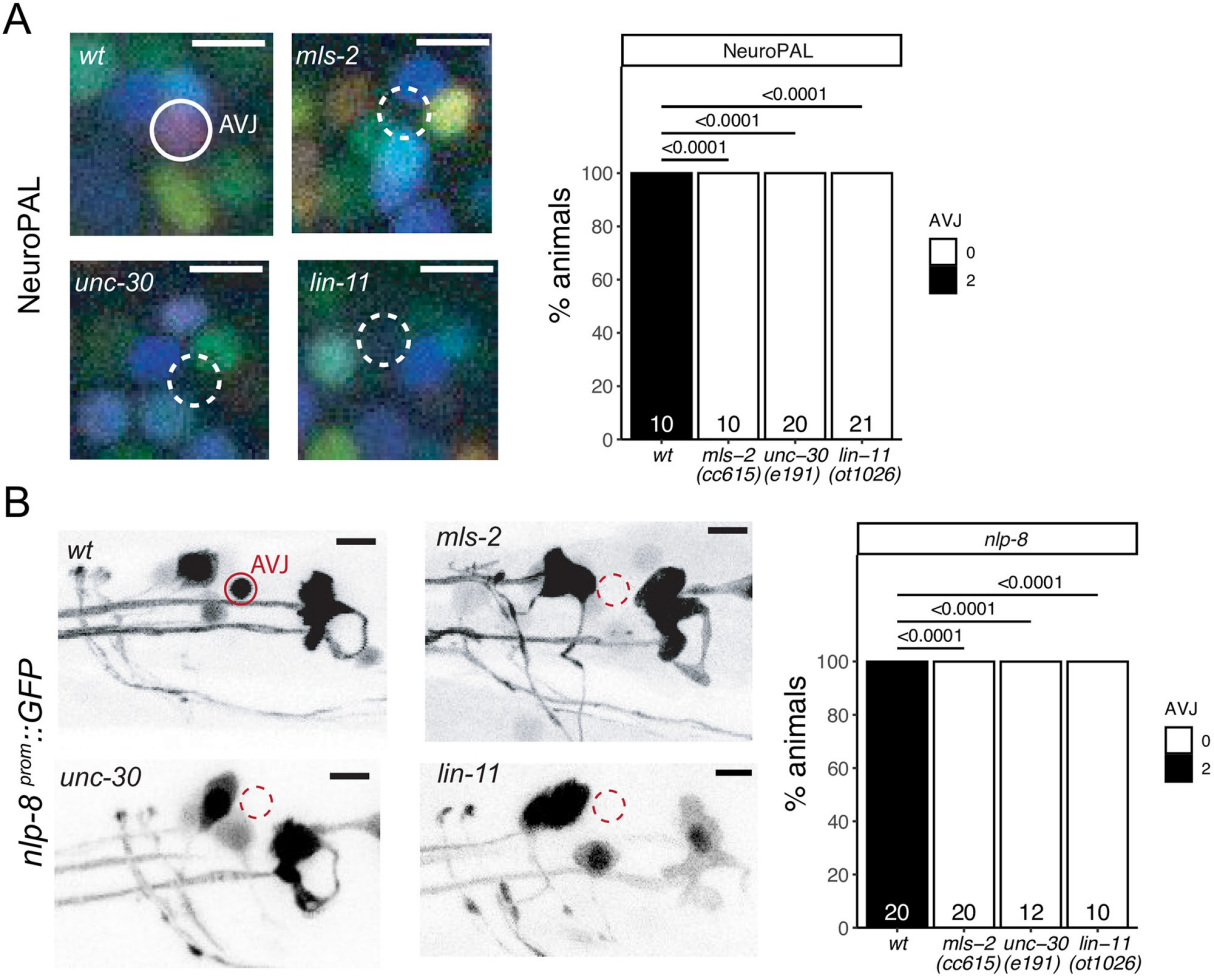
Three homeobox genes control the identity of the AVJ neuron class. **Fig 4A:** *mls-2 (cc615)*, *unc-30 (e191)* and *lin-11(ot1026)* mutant animals show defects in the expression of NeuroPAL (*otIs669*) in AVJ. Neuron of interest is outlined in solid white when expressing wildtype reporter colors, and dashed white when one or all colors are lost. Representative images of wildtype and mutant worms are shown with 5 μm scale bars. Graphs compare expression in wildtype worms with the number of animals examined listed at the bottom of the bar. P-values were calculated by Fisher’s exact test. **Fig 4B:** *mls-2 (cc615)*, *unc-30 (e191)* and *lin-11(ot1026)* mutant animals show defects in the expression of an *nlp-8* reporter transgene (*otIs711*) in AVJ. Neuron of interest is outlined in solid red when expressing wildtype reporter colors, and dashed red when one or all colors are lost. Representative images of wildtype and mutant worms are shown with 5 μm scale bars. Graphs compare expression in wildtype worms with the number of animals examined listed at the bottom of the bar. P-values were calculated by Fisher’s exact test.

Apart from the AIM interneuron class, *mls-2* is also continuously expressed in the AVJ neuron pair, an interneuron class in the lateral ganglion with no presently known function or identity regulator. The AVJ interneuron class appears morphologically very similar to its neighboring AVH neuron class, and AVJ has been commonly misidentified as AVH [33]. NeuroPAL provides a unique color code for AVJ, based on the expression of the neuropeptide-encoding *flp-26* gene and the AMPA Glu receptor *glr-1* [15,34]. *mls-2* affects expression of these genes, but panneuronal marker expression remains unaffected, consistent with a role of *mls-2* as a terminal selector of AVJ identity (Fig 4A). We further corroborated this notion by testing the expression of an additional neuropeptide gene, *nlp-8*, whose expression in AVJ was predicted by CeNGEN and confirmed with a reporter transgene (Fig 4B). We find proper *nlp-8* expression in AVJ to be affected by loss of *mls-2* as well (Fig 4B).

In addition to *mls-2*, the AVJ neurons co-express the LIM homeobox gene *lin-11* and the Pitx-type homeobox gene *unc-30* [6]. This homeobox code is unique for the AVJ neurons [6]. While *unc-30* and *lin-11* were previously known to control the identity of other neuron classes [35–37], their function in AVJ has not been previously examined. Since the previously used *lin-11* mutant alleles are not unambiguous molecular nulls [38], we generated such a null allele by deleting all coding sequences using the CRISPR/Cas9 genome engineering. Using NeuroPAL as a cell fate assessment tool, we found differentiation defects in *unc-30* and *lin-11* null mutants similar to those observed in *mls-2* mutant animals (Fig 4A). Both *unc-30* and *lin-11* also affect expression of *nlp-8* in AVJ (Fig 4B).

We also note that the AVJ gene battery shows an enrichment of phylogenetically conserved DNA binding sites of UNC-30 and MLS-2 [3], consistent with these factors acting as terminal selectors of neuron identity. LIN-11 binding sites are not specific enough to permit genome-wide analysis [3,39].

### The Eyeless/Pax6 ortholog *vab-3* is required for the differentiation of several anterior ganglion neurons

**Fig 5:**
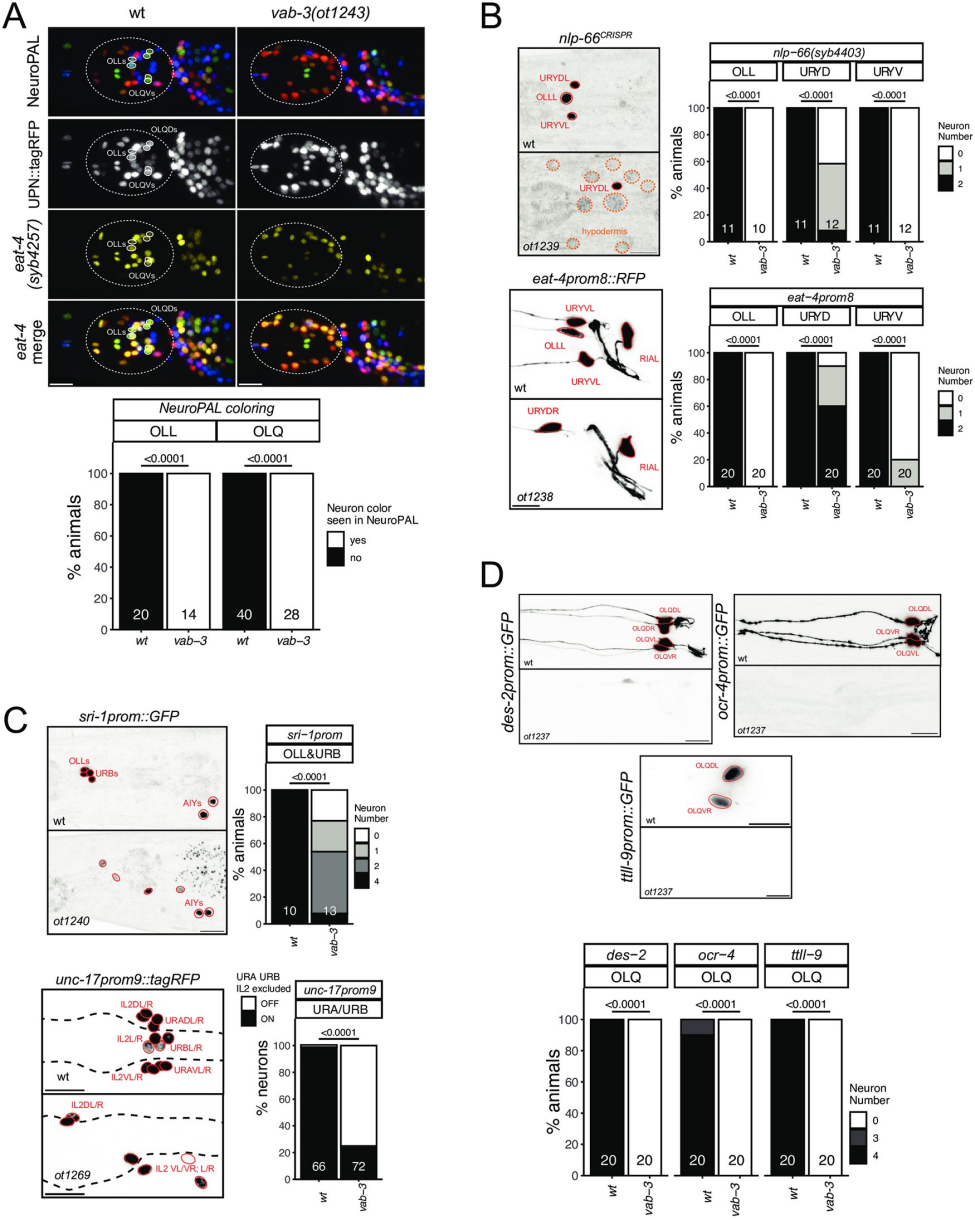
The Eyeless/Pax6 ortholog *vab-3* controls the identity of neurons in the anterior ganglion. **Fig 5A:** In a *vab-3(ot1243)* mutant allele, many neurons in the anterior ganglion lose their NeuroPAL coloring (from *otIs669)* and expression of the *eat-4* reporter allele *(syb4257)*. Notably, there are much less blue neurons (URY/URA/URB but URX seem present), and the bright green OLQ and turquoise OLL are never seen. Representative images of wild type and mutant worms are shown with 10 μm scale bars. Graphs compare expression in wild type and mutant worms with the number of neurons (for n = 10 WT worms / 7 *vab-3* mutant*)* examined listed at the bottom of the bar. P-values were calculated by Fisher’s exact test. Similar results were observed with a larger deletion allele, ot1269 (S7 Fig). **Fig 5B:** In *vab-3* mutant worms (*ot1239*, *ot1238*, all carrying the same lesion, introduced into respective reporter background; S7 Fig), the OLL and URYmarkers *nlp-66(syb4403)*, *eat-4prom8* (*otIs521*) are affected. Markers are more frequently lost in the ventral URY than the dorsal URY. *vab-3* mutants also ectopically express *nlp-66* in hypodermal cells. Representative images of wild type and mutant worms are shown with 10 μm scale bars. Graphs compare expression in wild type and mutant worms with the number of animals examined listed at the bottom of the bar. P-values were calculated by Fisher’s exact test. **Fig. 5C**: In *vab-3* mutant worms (*ot1240*, *ot1269;* S7 Fig), URA and URB identities are affected as seen with the markers *sri-1 (otIs879*) (URB, OLL) and a promoter fragment of *unc-17/*VAchT *(prom9; otEx7705)* [105] expressed in IL2/URA/URB. Representative images of wild type and mutant worms are shown with 10 μm scale bars. Graphs compare expression in wild type and mutant worms with the number of animals (*sri-1)* or neurons (*unc-17prom9)* examined listed at the bottom of the bar. *vab-3* does not affect expression of an *unc-17* reporter allele in the IL2 neurons, and we can therefore infer that the remaining positive cells labeled with *unc-17prom9*, are the IL2 neurons and the lost expression is in URA/URB. P-values were calculated by Fisher’s exact test. **Fig 5D:** Markers of OLQ neuron identity (*ocr-4 kyEx581*, *ttll-9 otIs850* and *des-2 otEx7697*) are fully lost in the *vab-3(ot1237)* mutant animals. Representative images of wild type and mutant worms are shown with 10 μm scale bars. Graphs compare expression in wild type and mutant worms with the number of animals examined listed at the bottom of the bar. P-values were calculated by Fisher’s exact test.

As described above (Fig 1), the Pax6/Eyeless ortholog *vab-3* [40] is continuously expressed in a number of differentiating neurons, from their birth throughout their lifetime. *vab-3* is most prominently expressed in many neuron classes of the most anteriorly located head ganglion, called the anterior ganglion (OLQ, URY, OLL, URA, URB, CEPV and, more strongly than in all other neurons, BAG). We have previously shown that *vab-3* is required for the proper differentiation of the OLL neurons and the ventral URY neurons (URYV) [32], while others have shown a role in BAG neuron differentiation [41]. Seeking to extend this past analysis, we first used NeuroPAL to assess the differentiation program of anterior ganglion neurons in *vab-3* mutant mutants. We note loss of neuron-type specific NeuroPAL color codes in many anterior ganglion neurons in *vab-3* mutants, but still observe panneuronal marker expression Fig 5A), indicating that neurons are generated, but fail to properly differentiate into specific types. We could confidently identify the glutamatergic OLL and OLQ neurons to have lost their proper color code (Fig 5A). Consistent with this, an *eat-4* reporter allele, expressed in all glutamatergic neurons in the anterior ganglion, shows loss of fluorescent signals in many neurons of this ganglion (Fig 5A).

Due to the overall disorganization of *vab-3* mutant heads, a consequence of epidermal morphogenesis defects [42], we sought to generate and examine markers that are more specifically, if not exclusively expressed in *vab-3(+)* neurons, so that marker loss could be more unambiguously assigned to a specific neuron class. To identify such markers, we made use of the scRNA CenGEN atlas [19]. A neuropeptide encoding locus, *nlp-66*, shows highly enriched and restricted scRNA expression in URY and OLL. CRISPR/Cas9 genome engineering was used to insert an SL2::gfp::H2B reporter cassette at the 3’end of the *nlp-66* locus. As predicted, this reporter allele showed strong expression in OLL and weaker expression in both dorsal and ventral URY neuron pairs (Fig 5B). Expression of this reporter is eliminated in *vab-3* mutant animals (Fig 5B). Intriguingly, *nlp-66*::*gfp* reporter allele expression can be observed in what appear to be epidermal cells of *vab-3* mutant animals (Fig 5B). Brandt et al. reported a similar ectopic expression of BAG markers *ets-5* and *flp-17* in epidermal cells of *vab-3* mutants [41]. OLL and URY also show defects in expression of the glutamatergic marker *eat-4* in *vab-3* mutants (Fig 5B). This observation corrects previous analysis in which we had used a non-integrated version of the same *eat-4* reporter construct, but had mistaken URY for OLQ [32].

According to the scRNA atlas [19], as well as previous reporter gene studies [43], the G-protein coupled receptor *sri-1* is expressed in the OLL, URB and AIY neurons. We find that a chromosomally integrated *sri-1* promoter fusion shows decreased expression in OLL and URB of *vab-3* mutant animals (Fig 5C). Similarly, cholinergic identity of the URB neuron (measured with *unc-17/VAchT* expression) is affected, as is cholinergic identity of the URA neurons (Fig 5C).

The CeNGEN scRNA atlas also identified genes with very restricted expression in another *vab-3(+)* neuron class, the four radially symmetric OLQ sensory neurons. For example, transcripts for the *ttll-9* gene, encoding a tubulin tyrosine ligase-like gene, appear to be exclusive to the OLQ neurons [19]. A reporter gene fusion using 500 bp of promoter sequences confirms OLQ-exclusive expression (Fig 5D). Expression of this reporter is completely lost in *vab-3* mutant animals (Fig 5D). The *ocr-4* gene, encoding a TRP channel, was previously reported to be exclusively expressed in the OLQ neurons [44] and we found this expression to be largely eliminated in *vab-3* mutants (Fig 5D). Lastly, in the course of dissecting the *cis*-regulatory control regions of the *des-2* locus, which encodes a degenerin-type ion channel [45], we isolated a 1.3 kb fragment that drives exclusive reporter expression in the OLQ neurons (Fig 5D). This transgene also fails to be expressed in *vab-3* mutant animals. We conclude that *vab-3* is required for the proper execution of most, if not all neuronal differentiation programs of the anterior ganglion in which *vab-3* is normally continuously expressed.

### The SIX3/6 ortholog *ceh-32* is also required for the differentiation of several anterior ganglion neurons

**Fig 6:**
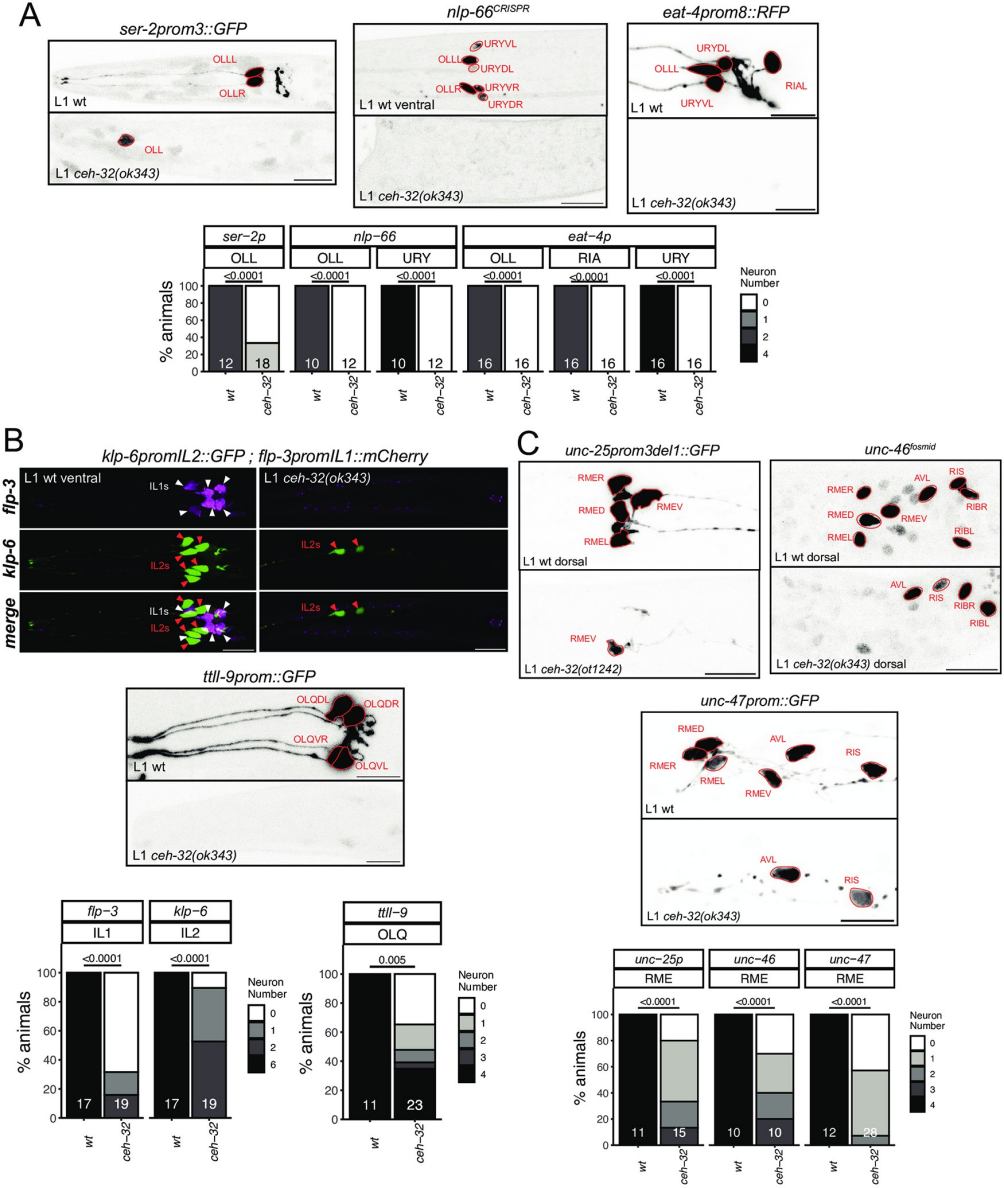
The SIX3/6 ortholog *ceh-32* controls the identity of neurons in the anterior ganglion. **Fig 6A:** In *ceh-32(ok343)* null mutant animals, OLL, URY but also RIA markers are almost always lost (*ser-2prom3 otIs138*, *nlp-66(syb4403)*, *eat-4prom8 otIs521)*. **Fig 6B:** *ceh-32* also controls IL1 identity (*flp-3* / *otIs703* marked with white triangles), and affects neurons where it is not expressed in adults (IL2: *klp-6 myIs13* marked with red triangles; OLQ: *ttll-9 otIs849*). Representative images of wild type and mutant worms are shown with 10 μm scale bars. Graphs compare expression in wild type and mutant worms with the number of animals examined listed at the bottom of the bar. P-values were calculated by Fisher’s exact test. **Fig 6C:** In *ceh-32(ok343)* and *ceh-32(ot1242)* null mutant animals, the GABAergic identity of all RME classes is affected (*unc-25prom3del1 otIs837*, *unc-47 oxIs12*, *unc-46* fosmid *otIs568*). *ceh-32* is expressed in adults in both RME and RIB, but *ceh-32* loss did not affect RIB identity (*unc-46 otIs568*). Representative images of wild type and mutant worms are shown with 10 μm scale bars. Graphs compare expression in wild type and mutant worms with the number of animals examined listed at the bottom of the bar. P-values were calculated by Fisher’s exact test.

The function of PAX and SIX homeobox genes is tightly intertwined in the context of development of anterior sensory structures, e.g. in eye development [46]. In *C*. *elegans*, *vab-3/PAX6* and *ceh-32/SIX3/6* function have been linked in the context of head morphogenesis [47]. The function of *ceh-32* in terminal neuron differentiation has only been reported in lateral ganglion neurons that express this gene (RIA, RMDD/V) [6, 18], but not in several anterior ganglion neuron classes in which *ceh-32* and *vab-3* are co-expressed throughout the neurons’ lifetime. We find that *ceh-32* phenocopies the differentiation defects of the OLL and URY neurons observed in *vab-3* mutants. Both *nlp-66* expression, as well as expression of the glutamatergic marker *eat-4* and the tyramine receptor *ser-2* are lost in the OLL and URY neurons of *ceh-32* mutants (Fig 6A).

The glutamatergic IL1 sensory/motor neurons, another *ceh-32(+)* neuron class in the anterior ganglion, also display differentiation defects in *ceh-32* mutants. Specifically, we find that expression of the neuropeptide gene *flp-3* is lost in the IL1 neurons (Fig 6B). A similar defect has been observed in *vab-3* mutants [48]; however, since *vab-3* is not expressed throughout IL1 development [6], its function may be restricted to earlier progenitor stages, perhaps upstream of *ceh-32*. A potential progenitor role is also evident for *ceh-32* in the OLQ and IL2 neuron classes. Both neuron classes fail to properly express differentiation markers in *ceh-32* mutants (Fig 6B). While there is no robust expression of *ceh-32* in postembryonic OLQ and IL2 neurons [6], *ceh-32* expression is evident in the embryonic lineage that generates both OLQ and IL2 neurons [20].

Apart from the mature anterior ganglion neuron classes OLL, URY and IL1, *ceh-32* is expressed in a number of additional postmitotic neurons, including the GABAergic RME motor neurons, which are also located in the anterior ganglion and which do not express *vab-3*. A nuclear hormone receptor, *nhr-67*, but no homeobox gene was previously shown to affect the identity of all RME neurons [49, 50]. Using three distinct markers of terminal GABAergic identity, *unc-25/GAD*, *unc-46/LAMP* and *unc-47/VGAT*, we find that *ceh-32* affects the identity acquisition of the RME neuron class (Fig 6C). GABAergic neuron identity was not affected in the RIB neurons of *ceh-32* mutant animals, which normally also express *ceh-32* (Fig 6C). Similarly, expression of the RIB-specific *sto-3* marker was also unaffected in the RIB neurons of *ceh-32* mutants. We conclude that *vab-3* and *ceh-32* are required for the proper terminal differentiation of a partially overlapping set of neurons in the anterior ganglion.

### *tab-1* functions in a lineage that produces the AIN and AVD neurons

**Fig 7:**
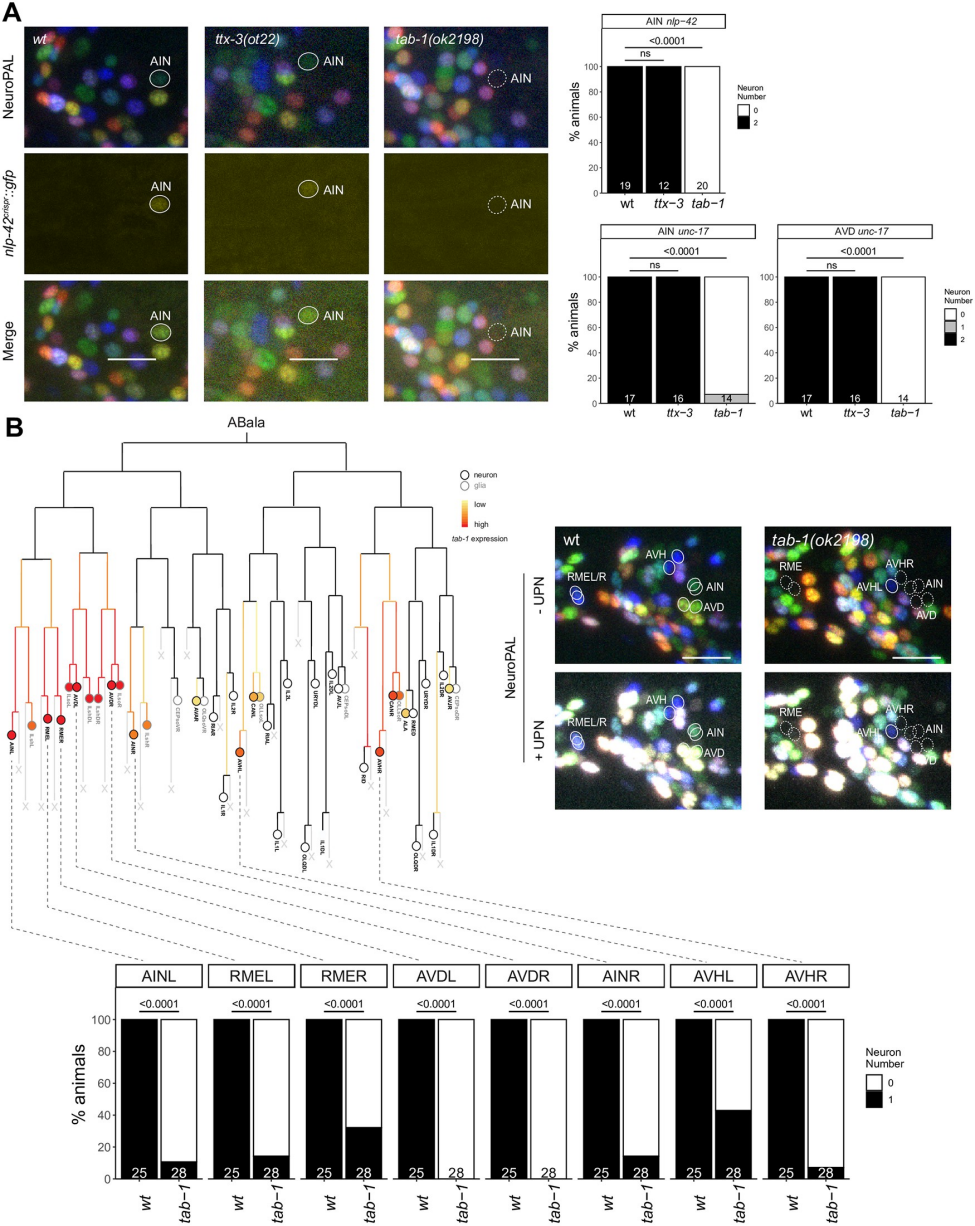
*tab-1* regulates the differentiation of various neurons in the ABala lineage. **Fig 7A:** In *tab-1(ok2198)* mutants, expression of both *nlp-42(syb3238)* and NeuroPAL reporters in AIN is lost. *tab-1(ok2198)* mutants also showed defects in *unc-17(otIs576)* reporter expression in AIN and AVD. No loss of reporter expression was observed in *ttx-3(ot22)* mutants. Representative images of wild type and mutant worms are shown with 10 μm scale bars. **Fig 7B:** *tab-1* is expressed in various neurons derived from the ABala lineage (adapted from Ma et al., 2021). In *tab-1(ok2198)* mutants, defects in NeuroPAL reporter expression, including ultrapanneuronal (UPN) reporter expression, are seen in neurons which express *tab-1* embryonically. Representative images of wild type and mutant worms are shown with 10 μm scale bars. In all panels, neurons of interest are outlined in solid white when expressing wildtype reporter colors, and dashed white when one or all colors are lost. P-values were calculated by Fisher’s exact test.

The cholinergic AIN interneuron class is another neuron class for which no identity specifier has been previously identified. The AIN neuron pair is labeled with three markers by NeuroPAL (*flp-19*, *cho-1* and *mbr-1*) and, being cholinergic, also expresses *unc-17/VAChT* [30]. In addition, the CeNGEN scRNA atlas reported very strong and selective expression of a neuropeptide, *nlp-42*, in AIN. We tagged the *nlp-42* locus with a T2A-based reporter cassette and confirmed selective expression in AIN (Fig 7). Armed with these markers we first considered the LIM homeobox gene *ttx-3*, which is continuously expressed in AIN [6, 14] and which is known to control the cholinergic identity of two other amphid interneuron (“AI”) classes, the AIY and AIA interneurons [12,14]. We found no obvious AIN differentiation defects in *ttx-3* mutants, as assessed with NeuroPAL, the *nlp-42* reporter allele and *unc-17* fosmid-based reporter (Fig 7A). Another homeobox gene expressed in the postmitotic AIN neurons is *tab-1*, a deeply conserved homeobox gene homologous to the *Drosophila* Brain-specific homeobox (Bsh) gene [51] and vertebrate Bsx genes [52]. We found that NeuroPAL as well as *nlp-42* and *unc-17/VAChT* signals were absent in the AIN neurons of *tab-1* mutants (Fig 7A). The only other cholinergic neuron that expresses *tab-1* postmitotically throughout their life is the AVD command interneuron class. The NeuroPAL and *unc-17/VAChT* signals are absent in the AVD neurons of *tab-1* mutants as well (Fig 7A).

Notably, the panneuronal signal from the NeuroPAL transgene is also not expressed in the AIN and AVD neurons of *tab-1* mutants, suggesting a proneural role of *tab-1*. The AIN and AVD neurons are generated from the ABalaa progenitor lineage [53] (Fig 7B). Embryonic expression of *tab-1* is observed very early throughout the entire ABalaa progenitor lineage [20] (Fig 7B), indicating that *tab-1* may have an early role in controlling progenitor fate in this lineage. Consistent with this notion, another class of neurons generated from the ABalaa lineage, the lateral RME neuron pair also require *tab-1* for their proper differentiation [50](Fig 7B). Outside the ABalaa lineage, *tab-1* is also expressed in neuroblasts that generate the AVH neurons, a neuron class that employs *unc-42* and *hlh-34* as terminal selectors [17]. These neurons also do not acquire any NeuroPAL color code in *tab-1* mutants (Fig 7B). Since *tab-1* is not continuously expressed in AVH [6], we again surmise an early, rather than late terminal differentiation/maintenance function of *tab-1*.

### The *egl-5* HOX gene is required for neuronal identity specification in head and tail neurons

**Fig 8:**
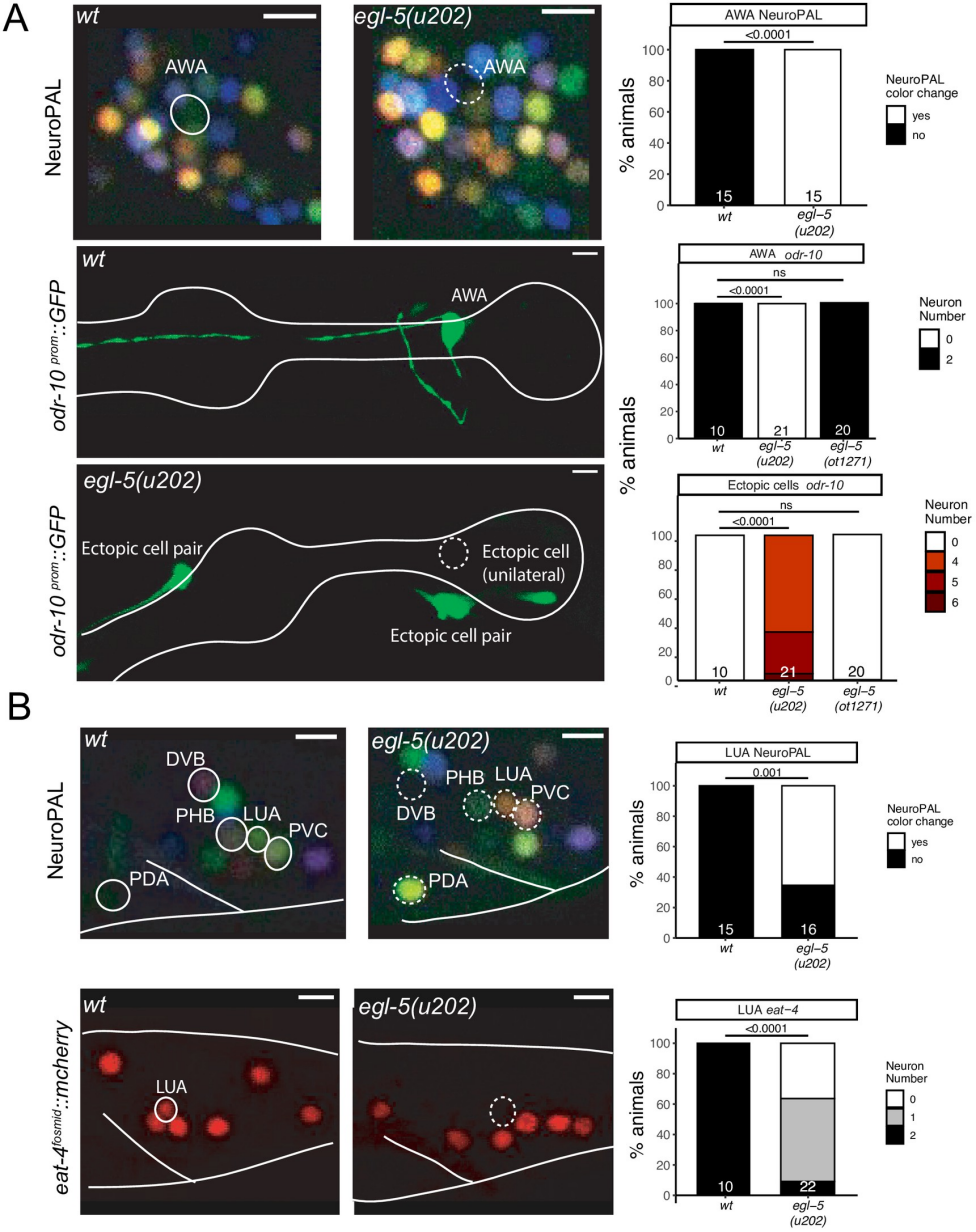
The HOX gene *egl-5* affects the differentiation of head and tail neurons. **Fig 8A:** *egl-5(u202)* mutant animals show a loss of AWA marker expression, including NeuroPAL (*otIs669*) and an *odr-10* reporter transgene (*kyIs37*). Representative images of wildtype and mutant worms are shown with 5 μm scale bars. Graphs compare expression in wildtype and mutant worms with the number of animals examined listed at the bottom of the bar. P-values were calculated by Fisher’s exact test. **Fig 8B:** *egl-5(u202)* mutant animals show changes in tail marker expression, including NeuroPAL (*otIs669*) in PDA, LUA, PVC and loss of eat-4 (*otIs518*) expression in LUA. Representative images of wildtype and mutant worms are shown with 5 μm scale bars. Graphs compare expression in wildtype and mutant worms with the number of animals examined listed at the bottom of the bar. P-values were calculated by Fisher’s exact test. In all panels, neurons of interest are outlined in solid white when expressing wildtype reporter colors, and dashed white when one or all colors are lost.

The expression of HOX cluster genes is traditionally thought to be absent in anterior cephalic structures [54]. We were therefore intrigued to observe expression of the *egl-5* HOX cluster gene, the *C*. *elegans* homolog of posterior Abdominal-B-type HOX genes that normally pattern posterior structures [55,56], in the AWA olfactory sensory neuron pair in the head of the animal [6]. The identity of this neuron was previously shown to be controlled by the *odr-7* nuclear hormone receptor, which regulates the expression of the olfactory receptor ODR-10 [57], as well as other identity features of AWA [3]. We found that in animals carrying the canonical *egl-5* allele *u202* [55], *odr-10* expression is also lost from AWA neurons and so is the characteristic NeuroPAL color code for AWA (Fig 8A). We instead observed ectopic expression of *odr-10* in other, positionally very distinct neuron classes in the head of *egl-5(u202)* mutants, indicating neuronal identity transformations (Fig 8A). However, these defects may be due to a neo- or antimorphic activity of *egl-5(u202)* since we did not observe *odr-10* expression defects in animals in which we deleted the entire *egl-5* locus using CRISPR/Cas9 genome engineering (Fig 8A).

Since our homeodomain protein expression atlas also detected the expression of two Otx-type homeodomain proteins in the AWA neurons, *ceh-36* and *ceh-37* [6], we tested whether they affect AWA differentiation, but found this not to be the case (S5 Fig). Also, *ceh-36* and *ceh-37* single mutants displayed no apparent defects in the differentiation of the ASI neurons, another neuron class in which our homeodomain protein expression atlas found these proteins to be expressed (S5 Fig).

As expected from its homology to AbdB, a posterior HOX gene, *egl-5* is also expressed in a number of neurons in the tail of the worm [6, 56, 58] and we find that *egl-5(u202)* affects the proper specification of a number of them. Specifically, the LUA interneuron, for which no previous identity regulator was known, shows defects in NeuroPAL color coding, as well as a loss of expression of the *eat-4* reporter allele (Fig 8B). NeuroPAL color code changes were also observed in the PDA and PHB neurons, suggesting that their identity is not properly executed either (Fig 8B). PDA and PHB differentiation defects of *egl-5(u202)* mutants were also recently reported [59], while this work was under preparation for publication. Other apparent NeuroPAL color changes in the tail of *egl-5* mutant animals are likely reflections of neuronal identity changes in the most posterior class member of ventral nerve cord motor neurons (e.g., DA9, VA12), that we previously reported with more cell-type specific markers in *egl-5* mutants [60] and that were corroborated by a more recent study [59].

### The RIC neuron class requires *unc-62/MEIS* for proper differentiation

**Fig 9:**
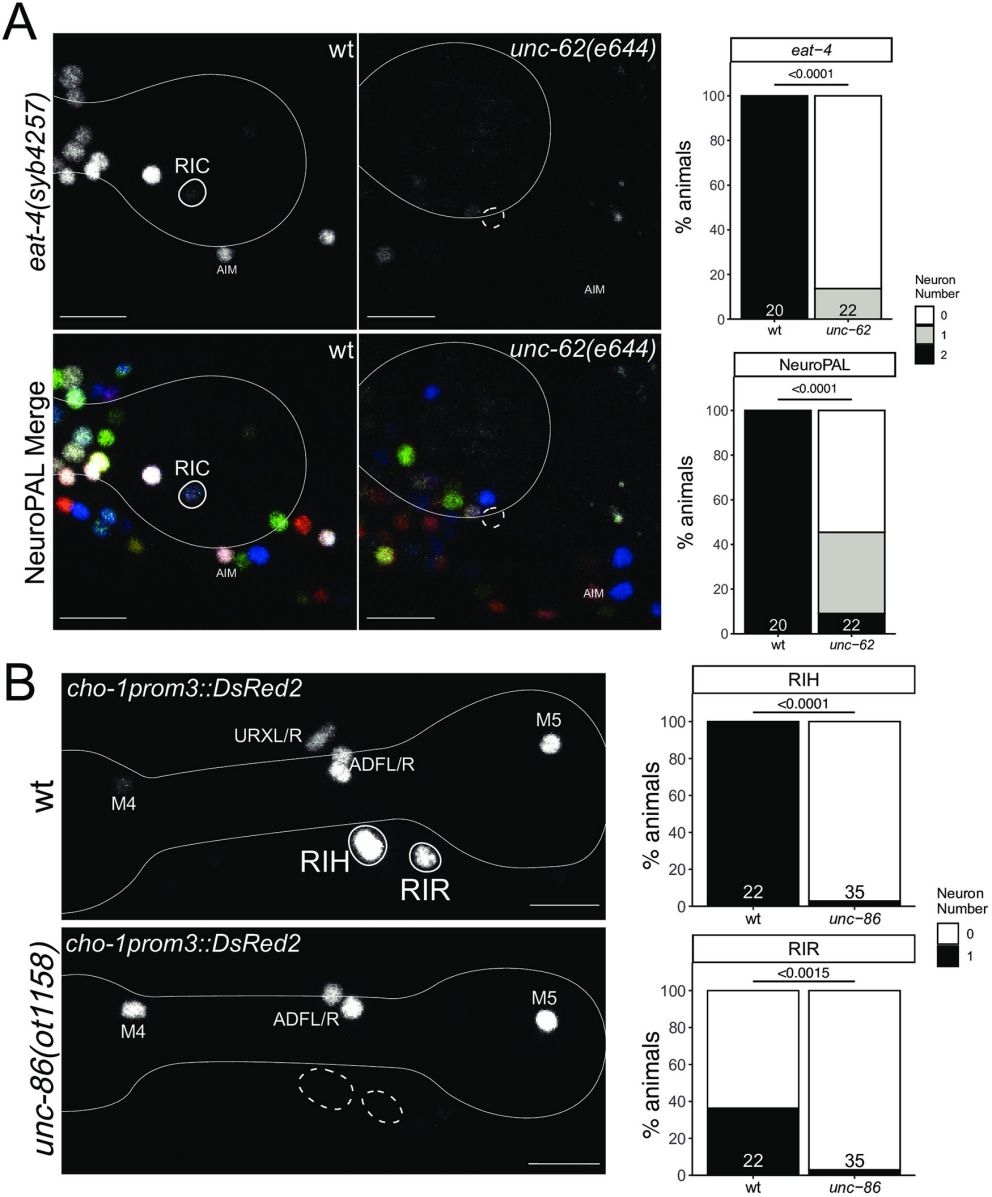
Ring interneuron (RIC, RIH, RIR) differentiation defects in homeobox gene mutants. **Fig 9A:** *unc-62(e644)* mutant animals show a loss of RIC marker expression, including an *eat-4* CRISPR reporter (*syb4257)* and NeuroPAL (*otIs669*). Representative images of wild type and mutant worms are shown with 10 μm scale bars. Graphs compare expression in wild type and mutant worms with the number of animals examined listed at the bottom of the bar. P-values were calculated by Fisher’s exact test. **Fig 9B:** *unc-86(ot1158)* mutant animals show loss of RIH and RIR marker expression of an extrachromosomal *cho-1prom3* reporter (*otEx4530*) [105]. Representative images of wild type and mutant worms are shown with 10 μm scale bars. Graphs compare expression in wild type and mutant worms with the number of animals examined listed at the bottom of the bar. In all panels, neurons of interest are outlined in solid white when expressing wildtype reporter colors, and dashed white when one or all colors are lost. P-values were calculated by Fisher’s exact test.

The ring interneuron (“RI”) class RIC is the only octopaminergic neuron class in *C*. *elegans* [61]. In addition to synthesizing octopamine (using the biosynthetic enzymes TBH-1 and TDC-1), we also found that RIC uses glutamate as a neurotransmitter. Our original mapping of the sites of *eat-4/VGLUT* expression had not identified detectable expression in RIC [32]. However, prompted by the detection of *eat-4/VGLUT* transcripts in our scRNA brain atlas [19], we used CRISPR/Cas9 to insert an SL2::gfp:H2B reporter cassette into the *eat-4/VGLUT* locus. This reporter allele shows the same cellular sites of expression as the previously published fosmid-based reporter, but also revealed expression in RIC (Fig 9A).

Neuronal differentiation defects of the RIC neurons were previously reported in animals lacking two non-homeobox genes, *zip-5* and *nhr-2* [62], but no regulator of the homeobox family was previously known. RIC expresses the MEIS homeobox gene *unc-62* [6]. We found that in *unc-62(e257)* mutants, *eat-4*::*gfp* expression in RIC is lost (Fig 9A). Moreover, the fate signature of the RIC neurons in NeuroPAL (combination of *ggr-3* and *mbr-1*) [15] is eliminated in *unc-62* mutants. Panneuronal marker expression is unaffected, indicating that *unc-62* does not control the generation, but rather the identity specification of the RIC neurons. We also note that expression of *tbh-1*, encoding a tyramine-beta hydroxylase involved in octopamine biosynthesis [61] is not affected in *unc-62(e257)* animals, but since this allele is a non-null allele (the null allele is early embryonic lethal; [63]), this lack of defect is difficult to interpret.

### The cholinergic RIH and RIR interneurons require the *unc-86* homeobox gene for proper differentiation

Unlike the bilaterally symmetric RIC neuron pair, two other ring interneuron classes, the RIH and RIR neurons, are unpaired single neurons. Both neurons have a branched main process that projects into both sides of the nerve ring, but are very distinct in synaptic connectivity [1] and molecular profile [19](S2 Fig). However, both neurons are cholinergic and express the POU homeobox gene *unc-86/BRN3* [64]. Previous work has shown that the serotonergic co-transmitter identity of RIH is controlled by *unc-86* [65]. Cholinergic neurotransmitter identity of RIH is also affected by loss of *unc-86* (Fig 9B) [30]. Similarly, the RIR neuron, previously entirely unstudied, also fails to express a key cholinergic neurotransmitter gene (*cho-1/ChT*) in *unc-86* mutants (Fig 9B). A neuropeptide that is expressed in RIR, *nlp-52*, is, however, not affected by *unc-86* (n = 12).

### Differentiation of the ring interneurons RIP and RIB is affected by the OTX-type homeobox gene *ttx-1*

**Fig 10:**
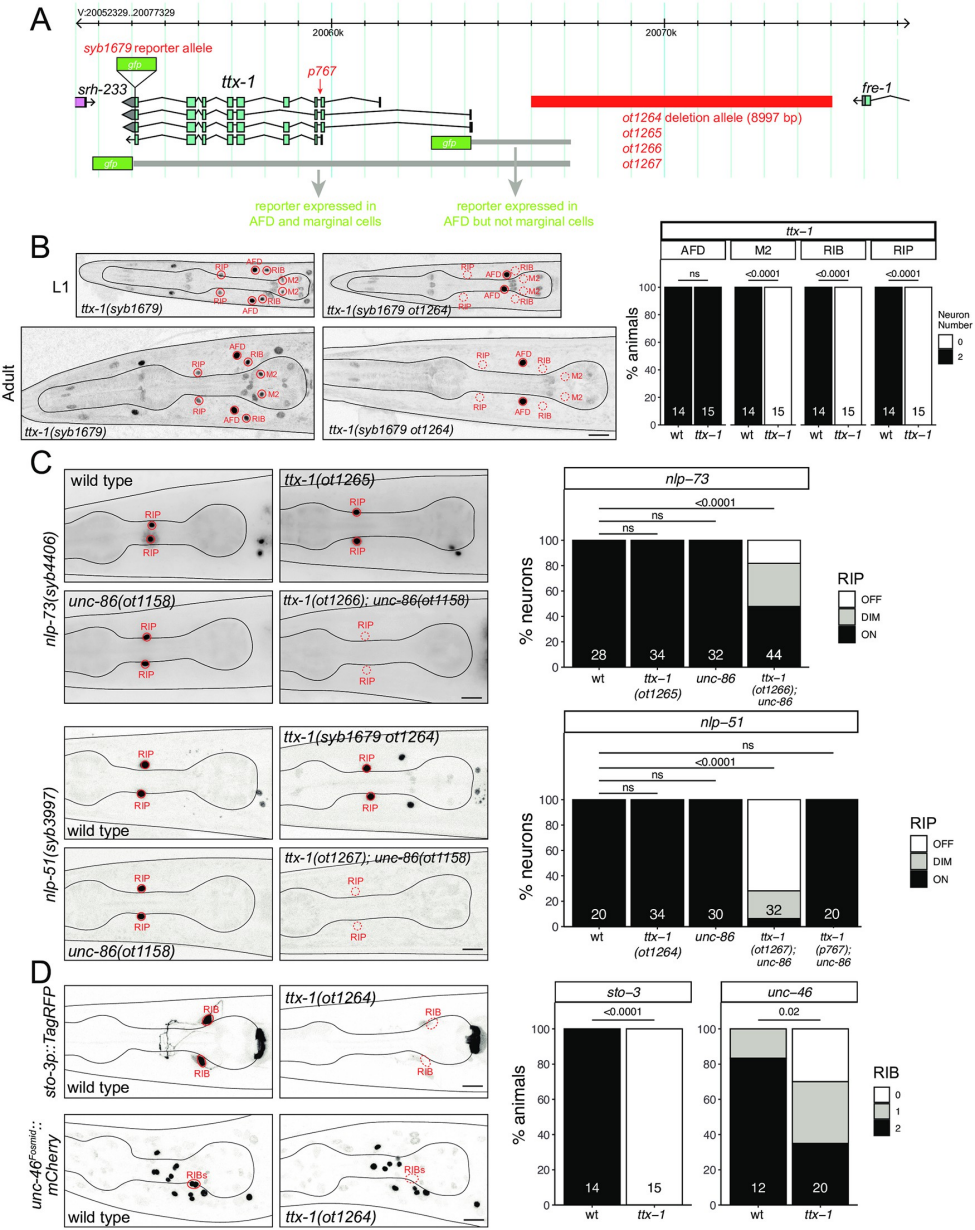
The OTX-type homeobox gene *ttx-1* affects the differentiation of the ring interneurons RIP and RIB. **Fig 10A:** *ttx-1* locus showing different alleles used in this study. **Fig 10B:** The *ttx-1(ot1264)* cis-regulatory allele loses *ttx-1(syb1679)* expression in RIP, RIB and M2 neurons. Representative images of wild type and mutant worms at the L1 and adult stage are shown with 10 μm scale bars. Graphs compare expression in wild type and mutant worms with the number of animals examined listed at the bottom of the bar. P-values were calculated by Fisher’s exact test. **Fig 10C:** *ttx-1* and *unc-86* show synergistic effects in RIP differentiation. Representative images of *nlp-73(syb4406)* and *nlp-51(syb3997)* reporter allele expression in wild type and mutant worms are shown with 10 μm scale bars. Graphs compare expression in wild type and mutant worms with the number of neurons examined listed at the bottom of the bar. P-values were calculated by Fisher’s exact test. **Fig 10D:** The *ttx-1(ot1264)* cis-regulatory allele affects RIB differentiation. Representative images of *sto-3 (otIs810)* and *unc-46 (otIs568)* reporter transgene expression in wild type and mutant worms are shown with 10 μm scale bars. Graphs compare expression in wild type and mutant worms with the number of animals examined listed at the bottom of the bar. P-values were calculated by Fisher’s exact test.

The RIP neurons are another ring interneuron pair that express *unc-86*. RIP is the only neuron that connects the main nervous system to the pharyngeal nervous system [1, 66], but both its function and its developmental specification have remained unexplored. Few molecular markers existed before the advent of the scRNA CeNGEN atlas. From the RIP-specific genetic signature that CeNGEN revealed [19], we selected two neuropeptide encoding genes that showed highly selective, strong transcript enrichment in RIP, the *nlp-51* and *nlp-73* genes, which aside from RIP, are also expressed in AIM (Fig 3C). CRISPR/Cas9 genome engineered *nlp-51* and *nlp-73* reporter alleles confirmed expression in RIP (Fig 10C). However, *unc-86* null mutants displayed no defects in *nlp-51* and *nlp-73* expression in RIP (Fig 10C).

We found that the Otx-type homeobox gene *ttx-1* is also expressed in RIP [6]. We first examined a previously described, viable splice site allele of *ttx-1*, *p767*, which displayed defects in AFD neuron differentiation [67]. In an *unc-86(ot1158); ttx-1(p767)* double mutant strain *nlp-51* expression remains unaffected (Fig 10C). However, since we found that a complete deletion of the *ttx-1* null locus results in embryonic lethality, the viable *p767* allele is not a null allele. To circumvent embryonic lethality, we sought to eliminate *ttx-1* function from the RIP neurons by generating *cis-*regulatory alleles of the *ttx-1* locus. Previous reporter analysis had located *cis*-regulatory elements that drive *ttx-1* expression in the AFD and the pharyngeal marginal cells to regions directly upstream of *ttx-1* (AFD) and the introns of *ttx-1* (marginal cells) [67](Fig 10A). Since the *ttx-1* reporter allele that we generated revealed additional sites of *ttx-1* expression in multiple neuron types in addition to AFD (RIP, RIB and M2) [6], we inferred that regulatory elements required for RIP, RIB and M2 expression are located more distally to the *ttx-1* start site. Using CRISPR/Cas9 genome engineering, we deleted 9 kb of sequences 2 kb upstream of the *ttx-1* start site, in a strain in which the *ttx-1* locus is tagged with *gfp* (Fig 10A). We found that in the resulting animals, *ttx-1(syb1679 ot1264)*, *ttx-1*::*gfp* expression in RIP, as well as the RIB and M2 neurons, is indeed eliminated (Fig 10B). While these animals still display no defects in *nlp-51 and nlp-73* expression in RIP, expression of both neuropeptides is now indeed reduced upon simultaneous removal of *unc-86* (Fig 10C). Similar redundant effects of *unc-86* with another homeobox gene have been identified in other neuron classes as well, e.g. the NSM neurons [7, 14].

Since the *cis-*regulatory allele of *ttx-1* also eliminates *ttx-1* expression in RIB, a GABAergic interneuron class with no previously known identity regulator, we assessed the expression of two markers of RIB identity, *unc-46* (involved in trafficking the vesicular GABA transporter) [68] and *sto-3* (a stomatin-like gene) [69]. We found that expression of both reporters is severaly affected in animals carrying the *ttx-1* cis-regulatory allele (Fig 10D).

### Homeobox genes affect the differentiation of neurons within the anterior deirid lineage

**Fig 11:**
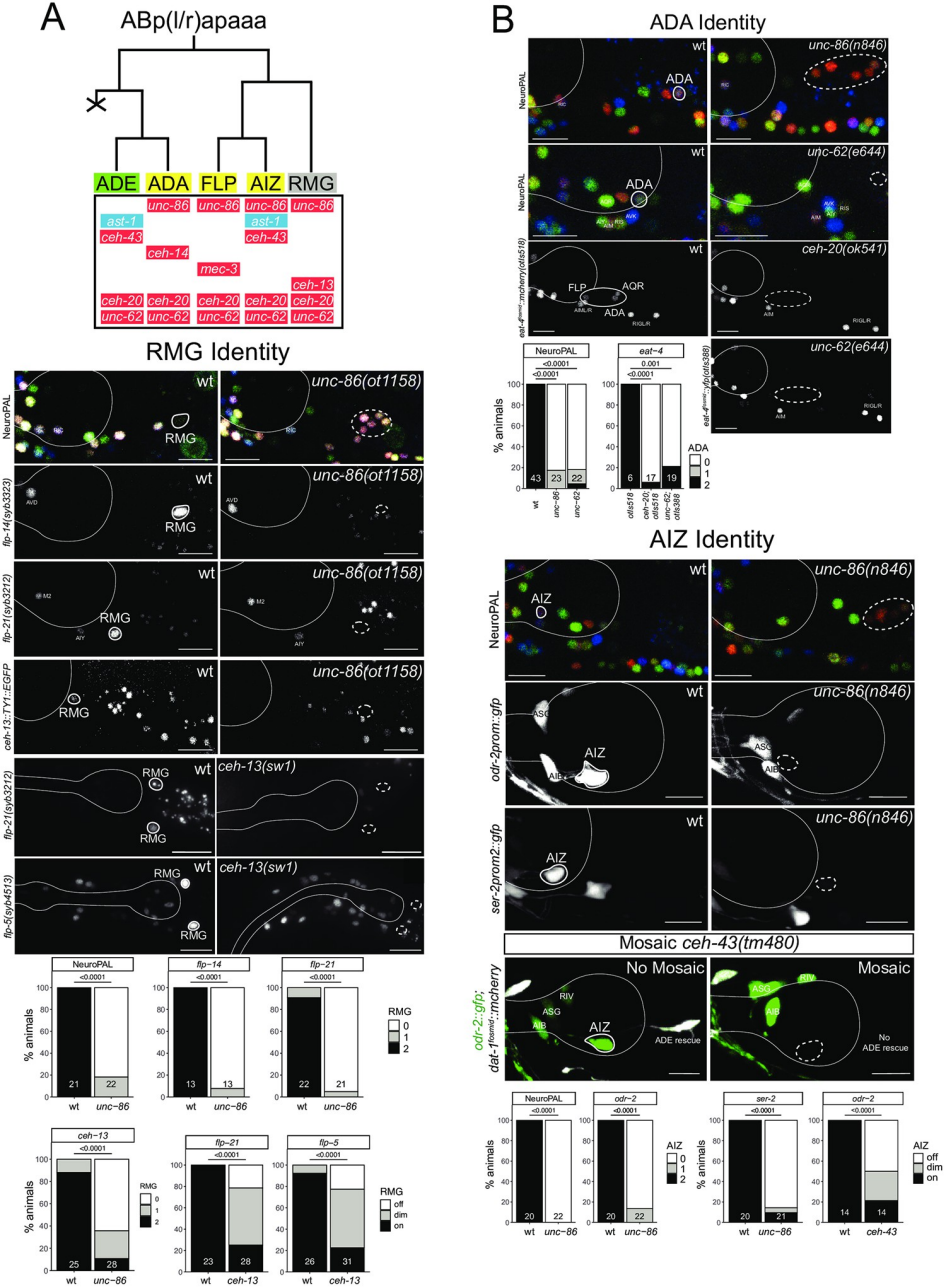
POU, MEIS and HOX genes control neuron identity in the anterior deirid lineage. **Fig 11A:** Lineage diagram depicting the generation of the anterior deirid neurons. Shown below each neuron in the lineage are the transcription factors known to be expressed in each of the respective neurons. Expression patterns for homeodomain proteins (all except AST-1) in the lineage are from [6, 91] and AST-1 is from this paper (S6 Fig). Green shading: dopaminergic neuron; yellow shading: glutamatergic neuron, grey shading: peptidergic neuron, red shading: homeobox gene, blue shading: non-homeobox gene. **Fig 11B:** Marker analysis in the anterior deirid lineage of wild-type and mutant animals. ADA Identity: *unc-62(e644)* and *unc-86(n846)* mutant animals show a loss of NeuroPAL (*otIs669*) in ADA. Additionally, *unc-86(n846)* mutant animals show a loss of an *eat-4* fosmid reporter (*otIs518*) in ADA. Representative images of wild type and mutant worms are shown with 10 μm scale bars. Graphs compare expression in wild type and mutant worms with the number of animals examined listed at the bottom of the bar. P-values were calculated by Fisher’s exact test. With two different *eat-4* fosmid based reporters, *unc-62* and *ceh-20* mutants also show differentiation defects in other neurons in the location of the anterior deirid, besides ADA (namely, FLP and AQR neurons). RMG Identity: *unc-86(ot1158)* mutant animals show a loss of RMG marker expression, including NeuroPAL (*otIs669*), a *flp-14* CRISPR reporter (*syb3323*), a *flp-21* CRISPR reporter (*syb3212*), and a *ceh-13* fosmid reporter (*wgIs756*). *ceh-13(sw1)* mutant animals show defects in the expression of two CRISPR reporters, *flp-5(syb3212)* and *flp-5(4513)*, in RMG. Representative images of wild type and mutant worms are shown with 10 μm scale bars. Graphs compare expression in wild type and mutant worms with the number of animals examined listed at the bottom of the bar. P-values were calculated by Fisher’s exact test. AIZ Identity: *unc-86(n846)* mutant animals show a loss of expression of NeuroPAL (*otIs669*) and an *odr-2* reporter transgene (*kyIs51*) and defects in the expression of a *ser-2* reporter transgene (*otIs358*) in AIZ. Representative images of wild type and mutant worms are shown with 10 μm scale bars. Graphs for NeuroPAL and *odr-2* compare expression in wild type and mutant worms with the number of animals examined listed at the bottom of the bar. Graphs for *ser-2* compare the brightness of expression (on, dim, off) in wild type and mutant worms with the number of animals examined listed at the bottom of the bar. In all panels, neurons of interest are outlined in solid white when expressing wildtype reporter colors, and dashed white when one or all colors are lost. P-values were calculated by Fisher’s exact test.

The glutamatergic, functionally and developmentally uncharacterized ADA neuron class is produced by the anterior deirid lineage (Fig 11A). The NeuroPAL transgene contains three molecular markers of ADA identity, *eat-4/VGLUT*, the acetylcholine receptor subunit *acr-5* and the neuropeptide *flp-26* [15]. The POU homeobox gene *unc-86/BRN3* is expressed in ADA [64] and given the role of *unc-86/BRN3* as terminal selector in several other neurons classes [70](this paper), we analyzed NeuroPAL expression in the ADA neurons of *unc-86* null mutants. We found that the NeuroPAL fate signature for ADA is lost in *unc-86* mutants (Fig 11B). Based on its continuous expression in all neurons of the anterior deirid, including ADA (Fig 11A), we considered the sole MEIS-type homeobox gene ortholog in *C*. *elegans*, *unc-62*, as a collaborator of UNC-86. Indeed, we observed the same NeuroPAL defects in the ADA neurons of *unc-62* mutants as we observed in *unc-86* mutants (Fig 11B). MEIS homeobox genes usually heterodimerize with PBX-type homeobox genes, of which there are three in *C*. *elegans* [63]. One of the three *C*. *elegans* PBX genes, *ceh-20*, is co-expressed with *unc-62* in all neurons of the anterior deirid lineage, including the ADA neurons [6](Fig 11A). Using an *eat-4/VGLUT* fosmid reporter, we found that ADA displays differentiation defects in both *unc-62* and *ceh-20* mutant animals (Fig 11B). At least one other glutamatergic neuron class in the anterior lineage (FLP), as well as the adjacent AQR neuron also fail to acquire glutamatergic identity (i.e. *eat-4/VGLUT* expression) in *unc-62/MEIS* and *ceh-20/PBX* mutants (Fig 11B).

Being part of the so-called anterior deirid lineage, the ADA neuron is lineally related to another interneuron whose differentiation program was previously uncharacterized, the RMG interneuron class (Fig 11A). RMG is a so-called “hub-and-spoke” neuron that integrates signals from various sensory neurons [71]. The RMG neuron pair expresses no classic, fast acting neurotransmitter pathway machinery, i.e. is neither cholinergic, glutamatergic, GABAergic or monoaminergic, but expresses a combination of neuropeptide-encoding genes [19, 29, 71]. Like ADA, the RMG neuron class also co-expresses *unc-86* and the Meis and Pbx homologs *unc-62* and *ceh-20* [6] (Fig 11A). Like in ADA, we find that *unc-86* is required for RMG differentiation, as assessed by its effect on neuropeptide gene expression (Fig 11B). Unlike ADA, the RMG neuron expresses the anterior most HOX cluster gene *ceh-13*, the *C*. *elegans* ortholog of Labial [6] (Fig 11A), which may act to distinguish ADA from RMG. We indeed found that *ceh-13* null mutant animals fail to properly express the neuropeptides *flp-5* and *flp-21* (Fig 11B).

Lastly, the DLX ortholog *ceh-43* is selectively expressed in two neuron classes of the anterior lineage, the dopaminergic ADE sensory neurons and the glutamatergic AIZ interneuron [6]. We had previously shown that *ceh-43* is required to specify ADE differentiation [13] and we find that loss of *ceh-43* also affects AIZ differentiation (Fig 11B).

### *unc-86* mutants display homeotic identity transformations in the anterior deirid lineage

Apart from ADA and RMG, other neurons within the anterior deirid lineage, specifically the AIZ and FLP neurons, also express the *unc-86/BRN3* homeobox gene (Fig 11A). Both neurons were previously shown to require *unc-86* for their proper differentiation [32,72]. This notion is further corroborated with individual cell-type specific markers, as well as the NeuroPAL transgene; all normally UNC-86(+) neurons in this lineage fail to acquire the proper color code in *unc-86* null mutants (Fig 11B). Notably, NeuroPAL reveals that these cells instead now adopt the color that is characteristic for the one non-UNC-86-expressing neuron in this lineage, the dopaminergic ADE neuron, marked with *dat-1* in the NeuroPAL transgene (Fig 11B). Upon the initial isolation of *unc-86* mutant alleles, additional cells with ectopic FIF staining, a chemical stain for dopamine, were noted in the anterior deirid lineage of *unc-86* mutant animals [73].

**Fig 12:**
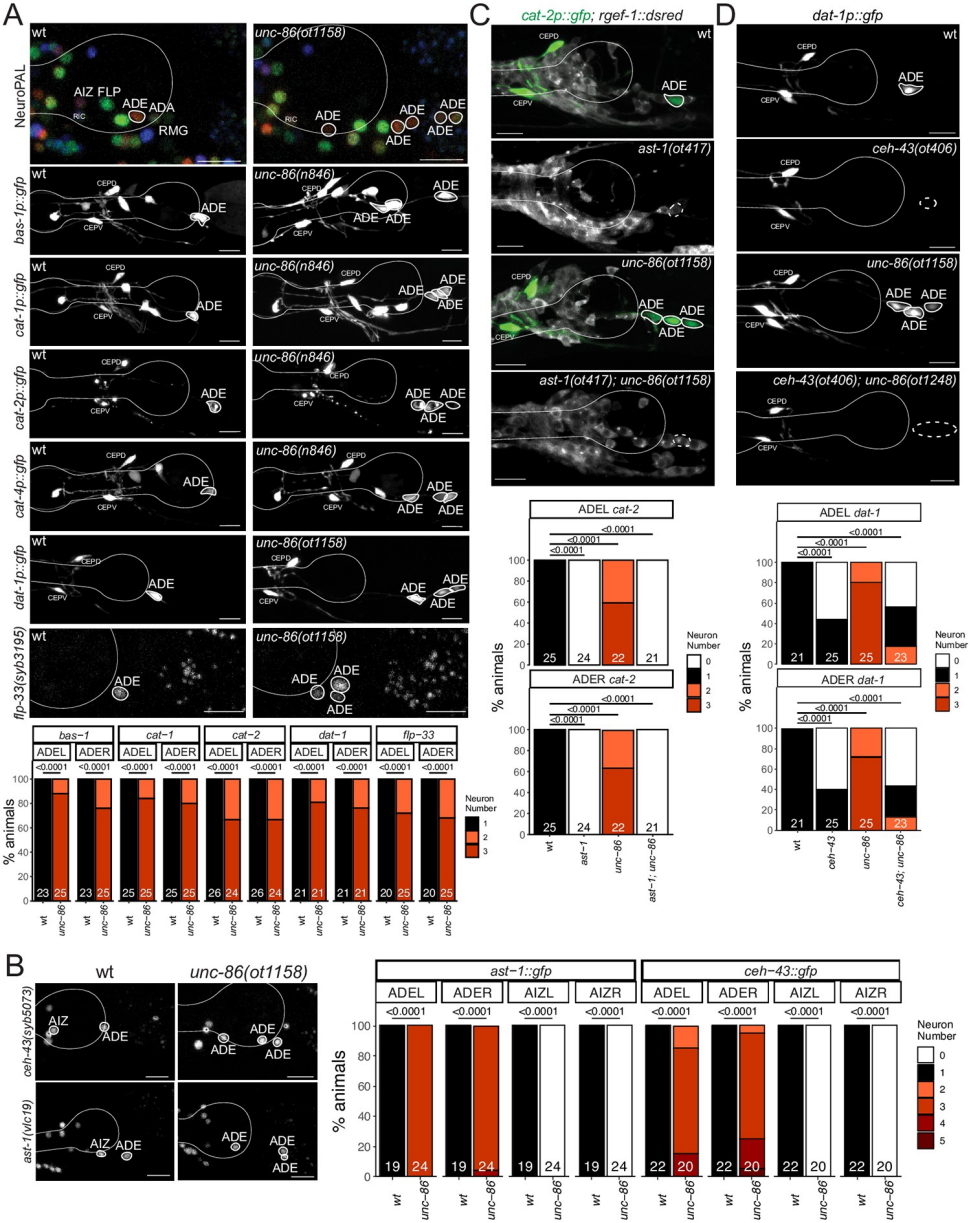
Derepression of dopaminergic terminal feature and dopaminergic regulatory signature in *unc-86* mutants. **Fig 12A:** *unc-86*(*ot1158*) and *unc-86(n846)* mutant animals ectopically express markers of ADE identity, including NeuroPAL (*otIs669*), reporter transgenes for genes involved in dopamine synthesis, including *bas-1 (otIs226)*, *cat-1 (otIs224)*, *cat-2 (nIs118)*, *cat-4(otIs225)* and *dat-1(vtIs1)*, and a *flp-33* CRISPR reporter (*syb3195*). Representative images of wild type and mutant worms are shown with 10 μm scale bars. Graphs compare expression on the left side (ADEL) and the right side (ADER) in wild type and mutant worms with the number of animals examined listed at the bottom of the bar. **Fig 12B:** *unc-86(ot1158)* mutant animals show a derepression of CRISPR/Cas9-engineered reporter alleles of *ceh-43(syb5073)* and *ast-1(vlc19)* in cells of the anterior deirid lineage. Representative images of wild type and mutant worms are shown with 10 μm scale bars (non-neuronal expression depicted with an asterisk). Graphs compare expression on the left side (ADEL and AIZL) and the right side (ADER and AIZR) in wild type and mutant worms with the number of animals examined listed at the bottom of the bar. **Fig 12C, FigD:** *ast-1* and *ceh-43* are epistatic to *unc-86*. The derepression of expression of the *cat-2* reporter transgene (*otIs199)* in *unc-86(ot1158)* mutant is suppressed in an *ast-1(ot417)* or an *ast-1(ot406)* mutant background. Note that both alleles are hypomorphic alleles (null alleles are lethal) [13,78]. Neurons of interest are outlined in solid white when expressing WT reporter colors, and dashed white when one or all colors are lost. Representative images of wild type and mutant worms are shown with 10 μm scale bars. Graphs compare expression on the left side (ADEL) and the right side (ADER) in wild type and mutant worms with the number of animals examined listed at the bottom of the bar. In all panels, neurons of interest are outlined in solid white when expressing wildtype reporter colors, and dashed white when one or all colors are lost and P-values were calculated by Fisher’s exact test.

We further probed the identity of the “ectopic” NeuroPAL/FIF-positive neurons by testing five markers of the dopamine biosynthesis and vesicular packaging pathway, *bas-1/AAAD*, *cat-1/VMAT*, *cat-2/TH*, *cat-4/GTPCH*, and *dat-1/DAT*. We found all genes to be ectopically expressed throughout the anterior deirid lineage of *unc-86* mutants (Fig 12A). Another marker for ADE fate that is independent of the dopamine biosynthesis pathway is the *flp-33* neuropeptide, which we also find to be ectopically expressed throughout the anterior deirid lineage of *unc-86* mutants (Fig 12A). Similarly, the *flp-5* gene, normally expressed in ADE, FLP and RMG also becomes expressed in more cells throughout the anterior deirid lineage.

We examined whether ectopic dopaminergic fate execution in the anterior deirid linage are a reflection of aberrant cell divisions leading to lineage reiterations, akin to those observed in the postdeirid lineage of *unc-86* mutants [73]. Using 4D embryonic lineage with the StarryNite package [74], we examined the cleavage patterns of six anterior deirid lineages (3 embryos) until the twofold stage in *unc-86* mutants and found no differences in cellular cleavage patterns between these mutants and wildtype animals.

Taken together, UNC-86 not only induces specific differentiation programs in cells of the anterior deirid lineage, but also represses an alternative neuronal identity (dopaminergic neurons) in these cells, which is normally executed by the only cell in the anterior deirid lineage that does not express *unc-86* (Fig 11A). Hence, *unc-86* appears to act as a “homeotic gene” whose loss results in neuronal identity transformations, akin to those observed in other genetic and cellular contexts in *C*. *elegans* [75–77].

### Derepression of a dopaminergic regulatory code in *unc-86* mutants

What is the underlying molecular basis for homeotic identity transformation in *unc-86* mutants? The terminal differentiation of all *C*. *elegans* dopaminergic neurons requires a combinatorial regulatory signature composed of at least four transcription factors, the ETS domain transcription factor *ast-1*, and the three homeobox genes *ceh-43/Dlx*, *unc-62/Meis* and *ceh-20/Pbx* [13,62,78](Fig 11A). *unc-62* and *ceh-20* are expressed in all neurons of the anterior deirid lineage (Fig 11A) and the data described above suggests that these factors are all required for each individual neuron fate in this lineage, apparently in combination with more restrictively expressed genes, namely *unc-86* in ADA, FLP, AIZ and RMG, and *ast-1* and *ceh-43* in ADE (Fig 11A). Consequently, in *unc-62* mutants, unlike in *unc-86* mutants, no ectopic dopaminergic fate is observed in the anterior deirid lineage (Fig 11B).

With this information in mind, an ectopic execution of dopaminergic fate throughout the anterior deirid lineage in *unc-86* mutants could mean that the complete regulatory signature for dopamine fate becomes de-repressed in *unc-86* mutants. We therefore considered the expression of both *ast-1* and *ceh-43* in the anterior lineage in wild-type and *unc-86* mutant animals. Our previous analysis of homeodomain protein expression showed that *ceh-43* is expressed in one other neuron of the anterior deirid lineage, the AIZ neuron, but it is not expressed in ADA, FLP or RMG [6](Fig 11A). We examined the expression of a reporter allele of *ast-1*, in which *gfp* has been inserted at the C-terminus of *ast-1* [79] and found that within the anterior deirid lineage *ast-1* shows the same expression pattern as *ceh-43*. Like *ceh-43*, *ast-1* is also expressed in AIZ, but not in ADA, FLP or RMG (Figs S6 and 11A). The dopamine regulatory signature therefore normally exists in AIZ, but *unc-86* apparently antagonizes the ability of *ast-1* and *ceh-43* to promote dopaminergic fate in AIZ, as inferred from AIZ normally not being dopaminergic, but turning on dopaminergic markers in *unc-86* mutants, as described above.

Furthermore, we observed that both *ceh-43* and *ast-1* expression becomes derepressed in additional neurons in the anterior deirid lineage in *unc-86* mutants (Fig 12). To ask whether derepression of this regulatory signature can indeed be made responsible for the dopaminergic identity transformation in *unc-86* mutants, we generated *unc-86; ast-1*, as well as *unc-86; ceh-43* double mutant animals. We found that in these animals, the generation of ectopic dopaminergic neurons is suppressed (Fig 12). Hence, we conclude that in the absence of *unc-86*, a regulatory signature of dopamine fate becomes derepressed throughout the anterior deirid lineage to permit induction of dopaminergic identity. Therefore, *unc-86* normally serves to antagonize dopaminergic fate specification, either by preventing the ability of *ast-1* and *ceh-43* to promote dopaminergic fate (in AIZ) or by preventing *ast-1* and *ceh-43* expression (in other cells in that lineage).

### Several homeobox gene mutants display no apparent neuronal differentiation defects

While neuronal differentiation defects are easily apparent in many homeobox gene mutants, we also note that several neuron types display no obvious differentiation defects in single homeobox gene mutants (S3 Table). However, absences of defects are not straight-forward to interpret for multiple reasons: First, because our cell fate marker analysis only usually tested a small number of markers, one cannot exclude that other markers would show a defect in expression. Limited marker analysis may also not be able to capture a cell identity transformation phenotype, particularly if the alternative differentiation program involves the expression of similar molecular markers (e.g. expression of the *eat-4/VGLUT* marker is unaffected in cases in which one glutamatergic neurons switches its fate to another glutamatergic neuron type). Second, in several cases only hypomorphic alleles could be analyzed since null alleles displayed phenotypic pleiotropies that complicated the assessment of neuronal differentiation defects. Third, in several previously documented cases, even unrelated homeobox genes from different subfamilies (e.g., LIM, POU) can act redundantly in neuron identity specification [7,14]. For example, neither *ttx-3* nor *unc-86* single mutants affect expression of several different differentiation markers of the NSM neurons, but in a *ttx-3; unc-86* double mutants these markers are completely lost [14]. A comprehensive analysis of neuronal cell fate in homeobox gene mutants may therefore require the generation of compound mutants.

## Discussion

Homeobox genes have been implicated in nervous system differentiation throughout animal phylogeny [5, 80–90]. What sets apart the nematode *C*. *elegans* from other model systems is the extent to which homeobox genes have been implicated in neuron identity specification throughout the entire nervous system. First, expression pattern analysis of homeodomain proteins has shown that each of the 118 anatomically defined *C*. *elegans* neuron classes not only expresses at least one homeodomain protein, but expresses a unique combination of them [6]. Second, homeobox genes have been shown to act as terminal selectors of neuron identity in many neuron classes of the *C*. *elegans* nervous system (reviewed in [35]). The first described case was the heteromeric UNC-86/MEC-3 homeodomain complex that coordinates the expression of terminal identity features of touch receptor neurons [11,91,92]. Other examples, covering many parts of the *C*. *elegans* nervous system, followed (reviewed in [35]). However, many neuron classes, while expressing homeobox gene combinations, had not previously been shown to require a homeobox gene for proper identity specification. Moreover, while many neuron classes were known to be affected by a specific homeobox gene, neuron-type specific homeobox co-factors had not yet been identified. For example, *unc-86* and *ceh-14* were both known to specify distinct neuron classes [30,32], but it remained to be shown whether another homeobox gene dictates their ability to control distinct gene batteries in different neuron classes.

In this work, we expanded our view of homeobox gene function in the *C*. *elegans* nervous system. As summarized in Table 2, the present analysis has identified functions for 14 homeobox genes in 24 different neuron classes that are functionally and lineally diverse and located in different parts of the nervous system. In 12 of these neuron classes no identity regulator was previously known; in 3 neuron classes only a non-homeobox gene was previously implicated in controlling terminal differentiation; and for 9 of these neuron classes homeobox genes were previously known to be involved in their differentiation, but our analysis has now added additional homeobox gene function, thereby providing unique neuron type-specific functional combinations. For example, *unc-86* and *ceh-14* function in the PHC versus the AIM neuron can now be, at least in part, explained by *unc-86* and *ceh-14* apparently cooperating with *mls-2* in the AIM neurons, but not the PHC neurons, where *mls-2* is not expressed.

Do the homeobox genes that we describe here act as master-regulatory terminal selectors? Criteria for such a role are that the protein (a) is continuously expressed during differentiation and throughout the life of the neuron to maintain the differentiated state; (b) coordinately controls the expression of many, and not just a small subset of identity features; (c) exerts this effect by direct binding to terminal effector genes. For our cell fate analysis, we often used the NeuroPAL transgene, which, depending on neuron class, contains multiple differentiation markers and/or we used a core, hardwired feature of neuronal identity, the neurotransmitter/neuropeptidergic phenotype of a neuron. In several cases described here, every single one of multiple markers is affected and it seems reasonable to extrapolate these defects on other identity markers as well. Moreover, we note that several of the homeodomain proteins that we functionally characterized here have known and distinctive DNA binding sites (based on either ChIP and/or protein binding microarray) and those are overrepresented in the gene batteries of neurons whose identity they control [3], arguing for coordinated control of the gene battery and hence arguing for a terminal selector role of these homeodomain proteins. For example, the transcriptome of the neurons that we show in this paper to require UNC-62/MEIS for proper differentiation (ADA, FLP, RMG, AQR) show an enrichment for predicted, phylogenetically conserved binding sites of UNC-62, as determined by a phylogenetic footprinting pipeline, TargetOrtho [3]. A similar enrichment of UNC-86 binding sites is observed in the *unc-86-*dependent neurons that we describe here and the same holds for MLS-2/HMX binding sites and CEH-14 binding sites in *mls-2-* or *ceh-14-*dependent neurons. Hence, we assume that in many of the cases we describe here, the respective homeodomain protein indeed serve as terminal selectors. However, we also note that in some cases, the respective homeobox gene is unlikely to act as a terminal selector. For example, the LHX3/4 ortholog CEH-14 affects only two out of four tested neuropeptide markers in the PVW neurons. In other cases too few markers were tested to confidently predict a terminal selector function.

**Fig 13:**
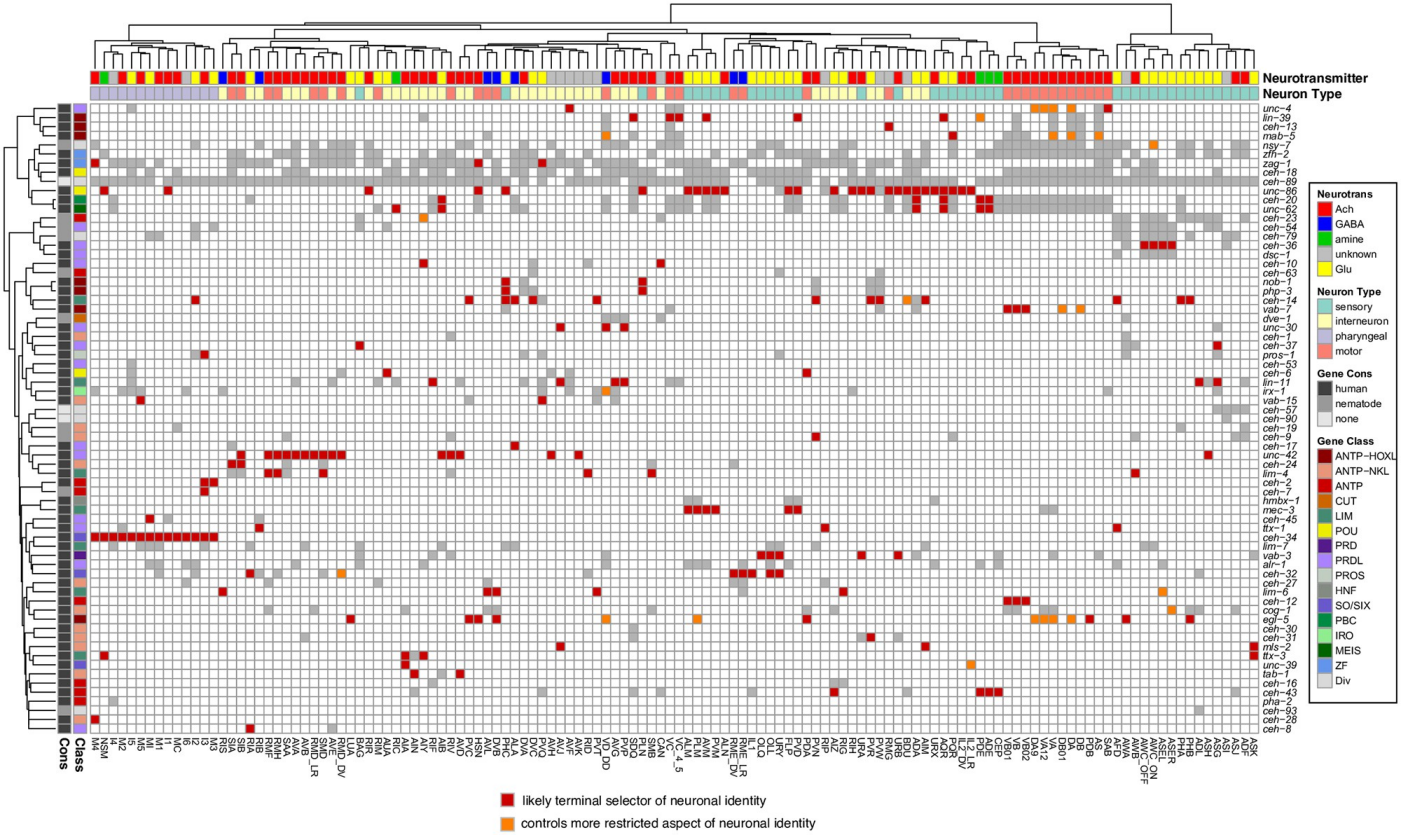
Regulation of neuron identity across the *C*. *elegans* nervous system by homeodomain transcription factors. Functional analysis of homeobox gene family, overlayed onto the homeobox expression matrix from Fig 1B. Red boxes indicate that a homeodomain transcription factor is expressed in and likely acts as a terminal selector for a given neuron type (based on extent of functional marker analysis). Orange boxes indicate that a homeodomain transcription factor has a more restricted function in a given neuron class. Gray boxes indicate that a homeodomain transcription factor is expressed in that given neuron type, but not necessarily functionally analyzed and white boxes indicate that a homeodomain transcription factor is not expressed in that given neuron type. Panneuronal and non-neuronal homeoboxes were excluded from this representation because they do not contribute to unique neuron type codes. Neuron types along the x axis are clustered by transcriptomic similarity using the Jaccard index (see methods) and homeobox genes along the y axis are clustered similarly by their similar expression profiles in shared neuron types. See S4 Table for tabular list of genes and cells on which this matrix is based.

No matter whether each homeobox gene coordinates neuronal identity as a terminal selector or only affects specific aspects of a neuron identity, in combination with previous analysis of homeobox gene function in the *C*. *elegans* nervous system, we can draw a striking conclusion: most, and perhaps all, of the 118 neuron classes of the *C*. *elegans* hermaphrodite require at least one postmitotically expressed homeobox gene to properly execute at least some aspect of their terminal differentiation program. Specifically, according to the current tally of homeobox gene analysis (depicted in Fig 13; listed in S4 Table), 113 of the 118 neuron classes now have a homeobox identity regulator assigned to them. This tally takes into account not only homeobox genes acting as terminal selectors, but also homeobox genes that may “only” act as subtype selectors (e.g. the *unc-4* gene, which diversifies VA from VB motor neuron fate, in combination with the non-homeobox gene *unc-3*) [93–96].

The 5 neuron classes that have not yet been found to require a homeobox gene for proper differentiation (ADF, ASI, ASJ, DVA, and RIM) do express combinations of homeobox genes, but their function still requires experimental validation. An assessment of the function of several of these homeobox genes is complicated by phenotypic pleiotropies (lethality) that several of these candidate genes display.

While in many cases, the loss of a homeobox gene results in the failure of a neuron to adopt an easily definable differentiated state, our analysis also provides a striking example of homeotic transformations in neuronal identity upon changes in homeobox codes. Specifically, we find that in the anterior deirid lineage, the POU homeobox gene *unc-86* promotes, in conjunction with neuron-type specific cofactors, the execution of several neuronal differentiation programs (FLP, ADA, AIZ, RMG), but also represses an alternative differentiation program. This alternative program–a dopaminergic neuron differentiation program–is normally promoted by two other terminal selectors, the Distalless/Dlx ortholog *ceh-43* and the ETS factor *ast-1*, but *unc-86* either represses their expression in some neurons of the anterior deirid lineage or antagonizes their function in one neuron class, the AIZ neurons, which normally express *ceh-43* and *ast-1*. One could imagine that a dopaminergic program may have been an “ancestral” state of several neurons in the anterior deirid, but the gain of *unc-86* expression in a subset of these cells diverted the differentiation programs of these cells into a different direction.

The homeotic transformation that we observe in the anterior deirid lineage of *unc-86* mutants is also observed in animals that lack the *lin-32* Atonal-like transcription factor [97]. *lin-32* has previously been shown to control *unc-86* expression in the anterior deirid lineage [98] and, hence, the phenotype of both *lin-32* and *unc-86* appear indistinguishable. Yet another homeotic cell identity transformation observed in *lin-32* mutants, an OLQ to URY identity transformation [97] can now also be explained with data presented here. Our updated expression, as well as functional data of *vab-3/PAX6* reveals that the gene is required for OLQ and URY specification, and we have shown here that *ceh-32/SIX* is required for URY identity specification. Derepressed *ceh-32* expression in OLQ observed in *lin-32/ATO* mutant [97], therefore generates a *vab-3 + ceh-32* combination in OLQ that is normally instructive for URY fate and, hence, leads to a switch from OLQ to URY identity. Our data therefore indicates that precise allocation of homeobox genes to specific cell types, achieved by upstream regulators such as *lin-32/ATO*, are critical to proper neuronal identity specification.

In several cases, we also infer dual roles of a homeobox gene early at progenitor stages, but also later in terminal differentiation. Such a potential dual role may be played by the BSH/BSX homolog *tab-1*, a homeobox gene that is already expressed early during several progenitor phases in the ABala lineage [20]. Loss of *tab-1* leads to an apparent failure to generate several neurons in this lineage. However, in a subset of these neurons (AIN and AVD), *tab-1* is not only expressed during progenitor stages but is also continuously expressed during terminal differentiation throughout embryonic, larval and adult development and may be involved in initiating and maintaining the differentiated state as well. A good precedent for such a scenario is the function of the *unc-86/BRN3* POU homeobox gene in the postdeirid lineage, where *unc-86* acts at a progenitor state to prevent the progenitor from continuing to divide, but then also later in a descendant of the progenitor, the PVD harsh touch sensory neuron, to initiate and maintain its differentiated state [64,73,99]. We note that embryonic homeobox gene expression data indicates that several homeobox genes are more broadly expressed during embryogenesis than they are in terminally differentiating nervous system [20, 100], suggesting that earlier progenitor roles remain to be discovered for a number of these genes. In one case, it has been found that such a role may only become evident upon removal of two apparently redundantly acting homeobox genes [101].

In conclusion, we interpret the preponderance of homeobox genes in terminal neuronal identity control in *C*. *elegans* to be an indication of homeobox genes having become recruited as neuronal identity regulators early in nervous system evolution [5]. To further probe this issue, a systematic analysis of homeobox gene function in other organisms should be in order, but such analysis will critically depend on (a) the ability to properly assess mutant phenotypes (which, in vertebrates, may involve the experimental prevention of the execution of cell death programs often triggered after neuronal misspecification; [99]) and (b) the ability to conditionally target genes to avoid confounding issues such as early patterning versus late differentiation roles.

Clearly, other families of transcription factors have acquired important roles in neuronal identity specification as well, both in *C*. *elegans* and in vertebrates. In *C*. *elegans*, non-homeobox genes often act together with homeobox genes to affect neuronal identity specification [35]. For example, the ETS domain transcription factor AST-1 cooperates with the UNC-86/BRN3 POU homeodomain transcription factor to define the fate of multiple distinct monoaminergic neuron classes [78,79]. However, no individual transcription factor family seems to be as broadly involved in neuronal identity specification as homeobox genes, even though they make up less little more than 10% of transcription factors in animal genomes.

## Material and methods

### Mutant strains and transgenes

A list of all strains and transgenes can be found in S5 Table. *vab-3*, *ceh-32*, *eya-1*, *unc-86*, *lin*-*11*, *egl-5* and *ttx-1* mutant alleles were generated using CRISPR/Cas9 and homology-directed DNA repair with a precise repair template [102]. All these alleles are shown in Figs S7 or 10A (*ttx-1*). For several loci the same genomic deletion was independently generated in different reporter backgrounds, resulting in different allele names (Figs S7 **and** 10A).

### CRISPR/Cas9 genome engineering

Reporter alleles were generated by CRISPR/Cas9 genome engineering inserting at the 3’end of the respective loci either a *gfp* tag (homeobox genes), an T2A::gfp::H2B cassette (*eat-4*), or, for neuropeptide-encoding genes, an SL2::gfp::H2B cassette or an T2A::3xNLS::gfp cassette. Reporter alleles were all generated by Sunybiotech. See S5 Table for each individual case. We generally found T2A::3xNLS::gfp to not give as bright a signal as other ensuing SL2::gfp::H2B cassette. We will report on systematic comparison between these cassettes elsewhere.

In addition to previously described reporter transgene (S5 Table), we generated a several additional transgene-based reporter construct. Promoter fusions for *des-2*, *ttll-9* and *sto-3* were Gibson-cloned into the pPD95.75 backbone, using either the SphI/XmaI multiple cloning sites. A PCR fusion reporter was generated for *sri-1* following [103] by Manasa Basavaraju. Primer sequences are:

*des-2*: 1.3kb promoter: **fwd** gttggctgacagatcaggtg **rev** cctgtagtaaaagtaaatgtgtgttgtgtg
*ttll-9*: 500bp promoter: **fwd** ACCAAGTTCGCTTATCAGTTG **rev** cacaacaaaaaaaatccaaaaactagtcg
sto-3: 974bp promoter: **fwd** gaggaatcatagatgcccaatcag **rev** aagccaaaccaagtgagaagaag
*sri-1*: 800bp promoter: **fwd** Gaaaattgcattatattaatgttgttcaag **rev** gagtttagcatactaaaaaATG

### Examination of expression reagents and neuron identification

For all homeobox genes where expression differences were noted relative to what was previously reported, we crossed the CRISPR/Cas9 generated reporter strain with the NeuroPAL landmark strain (*otIs669* or *otIs696*) and determined neuronal expression by colocalization of landmark colors and position of the neurons.

### Clustering of neuron types by transcriptomes

To cluster neurons by transcriptomic similarities (Figs S2, 1 and 12), we used the Jaccard index distance metric and clustered those distances using the Ward.D2 clustering method in R. The Jaccard distance metric is a binary determination of similarity between two groups. In this case, two neuron groups were assessed by the number of shared genes they express divided by the shared and unshared genes in those neuron groups. Thus, this analysis does not correlate levels of gene expression, but rather treats neuron types that express the same gene as similar. We then clustered those neurons based on their Jaccard distance using the Ward.D2 clustering method, which is agglomerative clustering mechanism where every neuron type starts in a separate cluster and is combined with similar neurons such that the internal cluster variance is minimized, until all neurons are part of a single cluster. We used the hclust() function in R to perform this analysis and the relationship of all neuron types is shown as a dendrogram.

Because each neuron type was sequenced at different depths there was an inconsistent number of transcripts picked up in each neuron type. Our initial clusterings using the complete transcriptome only showed relationships that reflected the depth of sequencing of each neuron type. To rectify this, we limited our transcriptomic analysis to only the top 500 expressed genes in each neuron type, assuming that those top genes were the important transcriptomic signature of that given neuron type. Therefore, our final clustering of the neuron types is based only on the 500 most highly expressed genes in each neuron type and whether or not those genes are similar to another neuron’s top 500 genes (S2 Fig).

### Mutant analysis scoring and statistics

Reporter expression was scored as an all-or-nothing phenotype. In some cases, the number of neurons were counted per animal as 0, 1, or 2 neurons for bilateral pairs, 0 through 4 neurons for 4-member neuron classes, and 0 through 6 for 6-member neuron classes. Other times the level of reporter expression was counted for each individual neuron as ON, DIM or OFF. For both cases scoring data was processed in R and converted as number of expressing neurons by genotype contingency tables. Statistical analysis was then done using the Fisher.test function in R under the two-sided null hypothesis. For our data, the Fisher.test function used the Mehta and Patel FEXACT algorithm to treat our data as multiple groups in a row by column contingency matrix and calculated the corresponding p-values for each group. The resulting adjusted p-values are shown as less than 0.0001 when appropriate. No statistical methods were used to determine sample size prior to experiment. Based on the common standard in the field, we aimed for n greater than or equal to 10 per genotype.

### Microscopy

Worms were anesthetized using 100mM of sodium azide (NaN_3_) and mounted on 5% agarose pads on glass slides. Images were acquired using confocal laser scanning microscopes (Zeiss LSM800 and LSM880) and processed using the ImageJ software [104]. For expression of reporters, representative maximum intensity projections are shown for GFP channel as gray scale and gamma and histogram were adjusted for visibility. For mutant functional analysis, representative maximum intensity projections are shown as inverted gray scale. NeuroPAL images provided in supplement are pseudocolored in accord with ^28^. All reporter reagents and mutants were imaged at 40x using fosmid or CRISPR reagents unless otherwise noted.

## Supporting information

S1 Fig Chromosomal location, reporters and summary of expression patterns of the two *C*. *elegans* BarH1 homologs, *ceh-30* and *ceh-31*. **A:** Comparison of expression of fosmid-based reporters (from [6]) to CRISPR/Cas9-engineered reporter alleles (see Fig 1 for expression pattern). The ubiquitous expression of the original *ceh-30* fosmid reporter may have been an artefact. SDQ expression of the *ceh-30* reporter allele *syb4678* may be a result of cis-regulatory elements upstream of *ceh-31*, which were not included in the *ceh-30* fosmid reporter, but were included in the *ceh-31* fosmid reporter (which produces an expression pattern identical to that of the reporter allele). **B:** Transcript abundance of *ceh-30* and *ceh-31* from the CeNGEN project, at 4 different threshold values [19]. Matches between scRNA data and homeodomain expression data are indicated in red. *ceh-31* transcripts completely match the sites of fosmid reporter (*wg370)* and reporter allele expression (*devKi250)*. *ceh-30* transcripts are indeed most strongly enriched in SDQ (the only neuron class where the reporter allele *syb4678* shows expression), but is observed in other neurons as well. While those do not show reporter allele expression, it is notable that they partially overlap with the sites of *ceh-31* expression. Perhaps these transcripts are produced from similar regulatory elements as the *ceh-31* transcripts, but are somehow not translated into protein.
(EPS)

S2 Fig Clustering of neuronal cell types based on molecular similarities. (EPS)

S3 Fig Numerical representation of homeobox expression data. This data uses the expression data from S1 and S2 Tables**.**
(EPS)

S4 Fig *eya-1* null mutant does not affect expression of AIA differentiation markers. We introduced the same CRISPR null deletion as in [7] in the *unc-17* and *flp-19* reporter alleles *syb4491* and *syb3278*, yielding alleles *ot1208* and *ot1209*. A small Z-stack (6–7 images at .8 micron) around the middle of the worm are shown with 10 μm scale bars. Graphs compare expression in wild type and mutant worms with the number of animals examined listed at the bottom of the bar. P-values were calculated by Fisher’s exact test.
(EPS)

S5 Fig Effect of loss of *ceh-36* or *ceh-37* on ASI, AWA, ASE markers. **A:** ASI and AWA analysis in *ceh-36(gj2127)* and *ceh-37(ok642)* mutant animals. Markers used are CRISPR/Cas9-engineered reporter alleles for *ins-3(syb5421)*, *ins-6(syb5463)*, *ins-24(syb5447)*, *ins-30(syb5526)*, *nlp-2(syb5697)* and the *odr-10(kyIs37)* reporter transgene. Representative images of wild type and mutant worms are shown with 10 μm scale bars. Graphs compare expression in wild type and mutant worms with the number of animals examined listed at the bottom of the bar. P-values were calculated by Fisher’s exact test. **B:** ASE analysis in *ceh-36(gj2127)* mutant animals. Markers used are CRISPR/Cas9-engineered reporter alleles for *ins-3(syb5421)*, *ins-30(syb5526)* and the *flp-6(ynIs67)* and *flp-13(ynIs37)* reporter transgenes. Representative images of wild type and mutant worms are shown with 10 μm scale bars. Graphs compare expression in wild type and mutant worms with the number of animals examined listed at the bottom of the bar. P-values were calculated by Fisher’s exact test.
(EPS)

S6 Fig Expression pattern of *ast-1(vlc19)* reporter allele. *ast-1* CRISPR/Cas9-engineered reporter allele, *vlc19*, [79] is expressed in the following head neuron classes: ADE, AIN, AIZ, ASG, AVG, CEPD, CEPV, I4, I5, M3, M5, RIV, RMD, RMDD, RMDV, SMBD, SMBV, SMDD, and SMDV. Expression in the midbody, ventral nerve cord, and tail was not examined.
(EPS)

S7 Fig Graphical representation of the homeobox mutant alleles that we generated by CRISPR/Cas9 genome engineering. Deletions were generated by CRISPR/Cas9 genome engineering using an oligo-repair template. Identical deletions introduced into different reporter strain backgrounds get separate allele names. For *lin-11*, the deletions *ot1025* and *ot1026* are the same, but *ot1241* is different by 4 nucleotides, even though the same oligo-mediated repair template was used.
(EPS)

S1 Table Homeodomain regulatory map, organized by homeodomain protein. This is an updated version of a table from [6].
(XLSX)

S2 Table Homeodomain regulatory map, organized by expressing neuron. This is an updated version of a table from [6].
(XLSX)

S3 Table Homeobox mutants showing no differentiation defects in specific neuron classes. (DOCX)

S4 Table A regulatory map of transcription factors with a role in terminal neuron differentiation. The basis for this data is taken mainly from [35] and supplemented with data from this paper, as well as others, including [6, 7, 17, 50, 59, 62, 79]. Criteria to be included in this list is that the transcription factor is expressed throughout embryonic and postembryonic development of the respective neuron type (i.e. is likely involved not only in initiation, but also maintenance of terminal differentiation programs) and the existence of mutant data that support a role in controlling marker genes. The homeobox part of this table is graphically presented in Fig 12.
(XLSX)

S5 Table List of strains used in this paper. (DOCX)
