# Supplementary material for: Widespread employment of conserved C. elegans homeobox genes in neuronal identity specification
Source: PLoS Genet. 2022 Sep 30;18(9):e1010372. doi: 10.1371/journal.pgen.1010372 (PMC9524666; doi:10.1371/journal.pgen.1010372)
Supplement: S5 Table — (DOCX) [file pgen.1010372.s012.docx]

**Supplementary Table S5: Strain list**

| **Strain Name** | **Genotype** | **DNA on array** | **Reference** |
| --- | --- | --- | --- |
| OH16745 | *vab-3(syb1653 ot1083) X* |  | This study |
| OH18007 | *vab-3(dev190([vab-3::mNeonGreen]); otIs669; him-5* | Bright NeuroPAL on V | [1] |
| OH18005 | *lim-7(devKi125 [mNeonGreen::lim-7]) I; otIs669; him-5 (e1490) V* | Bright NeuroPAL on V | [1] |
| PHX3426 | *ceh-27(syb2714[loxP] syb3286[loxP] syb3426[ceh27::GFP])* |  | This study |
| PHX5073 | *ceh-43(syb5073 [ceh-43::SL2::GFP::H2B])* |  | This study |
| PHX2880 | *ceh-16(syb2709[loxP] syb2880[ceh16::loxP::GFP])* |  | This study |
| PHX2934 | *ceh-36(syb2933[loxP]) ceh-36(syb2934[ceh36::loxP::GFP])* |  | This study |
| SYS629 | *ujIs113 (pie-1p::mCherry::H2B; nhr-2p::mCherry::his-24::let858 3’UTR; unc-119(+)) II; ceh-12 (devKi186 [ceh-12::mNeonGreen])* |  | [1] |
| SYS743 | *ujIs113 (pie-1p::mCherry::H2B; nhr-2p::mCherry::his-24::let858 3’UTR; unc-119(+)) II; unc-30 (hzhCR1 [unc-30::gfp])* |  | [1] |
| SYS608 | *ujIs113 (pie-1p::mCherry::H2B; nhr-2p::mCherry::his-24::let858 3’UTR; unc-119(+)) II; ceh-17 (devKi180 [mNeonGreen::ceh-17])* |  | [1] |
| SYS424 | *ujIs113 (pie-1p::mCherry::H2B; nhr-2p::mCherry::his-24::let858 3’UTR; unc-119(+)) II; ceh-5 (devKi103 [mNeonGreen::ceh-5])* |  | [1] |
| SYS397 | *ujIs113 (pie-1p::mCherry::H2B; nhr-2p::mCherry::his-24::let858 3’UTR; unc-119(+)) II; ceh-51 (devKi72 [mNeonGreen::ceh-51])* |  | [1] |
| SYS609 | *ujIs113 (pie-1p::mCherry::H2B; nhr-2p::mCherry::his-24::let858 3’UTR; unc-119(+)) II; ceh-82 (devKi181 [mNeonGreen::ceh-82])* |  | [1] |
| SYS635 | *ujIs113 (pie-1p::mCherry::H2B; nhr-2p::mCherry::his-24::let858 3’UTR; unc-119(+)) II; ceh-28 (devKi192 [mNeonGreen::ceh-28])* |  | [1] |
| SYS606 | *ujIs113 (pie-1p::mCherry::H2B; nhr-2p::mCherry::his-24::let858 3’UTR; unc-119(+)) II; ceh-2 (devKi17 [mNeonGreen::ceh-2])* |  | [1] |
| SYS634 | *ujIs113 (pie-1p::mCherry::H2B; nhr-2p::mCherry::his-24::let858 3’UTR; unc-119(+)) II; ceh-45 (devKi191 [mNeonGreen::ceh-45])* |  | [1] |
| SYS427 | *ujIs113 (pie-1p::mCherry::H2B; nhr-2p::mCherry::his-24::let858 3’UTR; unc-119(+)) II; ceh-10 (devKi101 [mNeonGreen::ceh-10])* |  | [1] |
| OH17991 | *otIs669; him-5 (e1490) V; ceh-31 (devKi250 [mNeonGreen::ceh-31]) X* | Bright NeuroPAL on V | [1] |
| MT19075 | *nIs352* | *eya-1p::gfp::eya-1* | [2] |
| OH17872 | *eya-1(ot1208) I; unc-17(syb4491[unc-17::T2A::GFP:H2B]) IV* |  | This study |
| OH17873 | *eya-1(ot1209) I; flp-19(syb3278 [flp-19::T2A::3×NLS::GFP]) X* |  | This study |
| PHX4678 | *ceh-30(syb4678[ceh-30::GFP])* |  | This study |
| OH17965 | *lin-11(ot1241);otIs711;; him-5* | *nlp-8prom::GFP, lin-15(+)* | This study |
| OH17870 | *lin-11(ot1026);unc-17(syb4491);otIs669* |  | This study |
| OH17869 | *lin-11(ot1025); nIs107; zfIs1010* | *tdc1::mcherry; tbh-1::GFP* | This study |
| CB845 | *unc-30(e191)* |  | [3] |
|  | *egl-5(u202)* |  | [4] |
| LW227 | *mls-2(cc615)* |  | [5] |
| OH15655 | *otIs711* | *nlp-8prom::GFP; lin-15(+)* | [6] |
| OH13645 | *otIs518* | *eat-4fosmid::mcherry; him-5* | [7] |
| OH11124 | *otIs388* | *eat-4fosmid::sl2::yfp::H2B* | [7] |
| CX3260 | *kyIs37* | *odr-10::GFP; lin-15(+)* | [8] |
| OH17950 | *vab-3(ot1237)* |  | This study |
|  | *oxIs12* | *unc-47prom::GFP* | [9] |
| PHX1656 | *ceh-8(syb1656[ceh-8::GFP])* |  | [10] |
| OH1422 | *otIs138* | *ser-2(promoter3), rol-6(su1006)* | [11] |
| OH16938 | *pha-1(e2123) otEx7697* | *des-2pOLQ::GFP, unc-122p::GFP, pha-1(+)* | This study |
| OH17344 | *otIs849* | *ttll-9p(500 bp)::GFP, pha-1(+)* | This study |
| OH17345 | *otIs850* | *ttll-9p(500 bp)::GFP, pha-1(+)* | This study |
| CX5478 | *lin-15(n765) kyEx581* | *ocr-4::GFP, lin-15(+)* | [12] |
| OH12525 | *otIs521* | *eat-4prom8::tagRFP; ttx-3prom::gfp* | [13] |
| OH17951 | *vab-3(ot1238); otIs521* | *eat-4prom8::tagRFP; ttx-3prom::gfp* | This study |
| OH15034 | *otIs653* | *srg-8promASK cho-1promAIA GRASP* | [14] |
| OH17963 | *otIs879* | *sri-1prom::NLS::GFP, pha-1(+)* | This study |
| OH17964 | *vab-3(ot1240); otIs879* | sri-1prom::NLS::GFP, pha-1(+) | This study |
| OH17124 | *otIs837* | *otIs837 [unc-25(prom3)(del1)::GFP* | [15] |
| OH17970 | *ceh-32(ot1242)/+; otIs837* | *otIs837 [unc-25(prom3)(del1)::GFP* | This study |
| PHX4491 | *unc-17(syb4491[unc-17::T2A::GFP:H2B])* |  | [6] |
| PHX4257 | *eat-4(syb4257[eat-4::T2A::GFP::H2B])* |  | [6] |
| OH17971 | *eat-4(syb4257[eat-4::T2A::GFP::H2B]) vab-3(ot1243); otIs669* | Bright NeuroPAL on V | [6] |
| PHX4403 | *nlp-66(syb4403[nlp-66::SL2::gfp::H2B])* |  | This study |
| OH17952 | *nlp-66(syb4403[nlp-66::SL2::gfp::H2B])*  *vab-3(ot1239) X* |  | This study |
| OH15338 | *ceh-32(ok343); otEx7146* | *ceh-32fosmid WRM0637dA10 ;myo-2::RFP* | [16] |
| OH15601 | *myIs13; otIs703* | *[klp-6prom::GFP]* *[flp-3prom::mCherry]* | [16] |
| OH13026 | *otIs568* | *unc-46fosmid::SL2::H2B::mCHOPTI; pha-1 rescue* | [17] |
| PHX3278 | *flp-19(syb3278 [flp-19::T2A::3×NLS::GFP])* |  | [16] |
| *PHX5452* | *ins-1(syb5452[ins-1::SL2::gfp::H2B])* |  | [16] |
| PHX4514 | *dmsr-2(syb4514 [dmsr-2::SL2::gfp::H2B])* |  | [16] |
| OH17241 | *unc-86(ot1158)* |  | [16] |
| CB257 | *unc-39(e257)* |  | [3] |
| OH15363 | *him-5(e1490); otIs669* | Bright NeuroPAL on V | [18] |
| OH17491 | *him-5(e1490) unc-39(ot1173); otIs669* | Bright NeuroPAL on V | [16] |
| MT1859 | *unc-86(n846)* |  | [19] |
| CB644 | *unc-62(e644)* |  | [3] |
| GJ3758 | *che-1(ot856); ceh-36(gj2127)* |  | [20] |
| NFB1369 | *ast-1(vlc19[ast-1::gfp])* |  | [21] |
| OH8251 | *otIs226* | *bas-1prom::gfp* | [22] |
| OH8249 | *otIs224* | *cat-1prom::gfp* | [23] |
|  | *nIs118* | *cat-2prom::gfp* | [24] |
| OH8250 | *otIs225* | *cat-4prom::gfp* | [23] |
| BY200 | *vtIs1* | *dat-1prom::gfp* | [25] |
| PHX3195 | *flp-33(syb3195[flp-33::T2A::3xNLS::GFP])* |  | This study |
| OH7197 | *ast-1(ot417); otIs199[cat-2prom::gfp; rgef-1::dsred]* |  | [22] |
| OH7323 | *ceh-43(ot406); vtIs1; vsIs33* |  | [23] |
| OH18009 | *unc-86(ot1248); ceh-43(ot406); vtIs1; vsIs33* |  | This study |
| PHX4257 | *eat-4(syb4257[eat-4::T2A::GFP::H2B])* |  | [26] |
| OH15422 | *ceh-14(ot900)* |  | [27] |
| OH10210 | *otEx4530* | *cho-1prom3::DsRed2* | [13] |
| OH10765 | *otIs358* | *ser-2prom2::gfp; pha-1(e2123)* | [28] |
| CX3300 | *kyIs51* | *odr-2prom::gfp* | [29] |
| OP756 | *unc-119(tm4063) III; wgIs756 [ceh-13::TY1::EGFP::3XFLAG + unc-119(+)]* | *ceh-13* fosmid reporter | [30] |
| PHX3323 | *flp-14(syb3323[flp-14::T2A::3xNLS::GFP])* |  | [16] |
| PHX3212 | *flp-21(syb3212[flp-21::T2A::3xNLS::GFP])* |  | This study |
| PHX4513 | *flp-5(syb4513[flp-5::SL2::GFP::H2B])* |  | [16] |
| FR431 | *ceh-13(sw1)/qC1 dpy-19(e1259) glp-1(q339) III* |  | [31] |
| KRA758 | *ceh-13(sw1)/hT2 III; flp-21(syb3212 [flp-21::T2A::3×NLS::GFP]) V* |  | This study |
| KRA759 | *mab-5(e1239) III; flp-6 (syb3203 [flp-6::T2A::3xNLS::GFP])V* |  | This study |
| KRA760 | *mab-5(e1239) III; nlp-40(syb3208 [nlp-40::T2A::3xNLS::GFP])I* |  | This study |
| KRA761 | *mab-5(e1239) III; otIs92[flp-10::gfp]V* |  | This study |
| OH17006 | *otIs576; otIs669; him-5(e1490) V* | *otIs576* [*unc-17 fosmid::GFP; lin-44::YFP*]  *otIs669:* NeuroPAL | This study |
| OH17049 | *tab-1(ok2198) II; otIs576; otIs669; him-5(e1490) V* | *otIs576* [*unc-17 fosmid::GFP; lin-44::YFP*]  o*tIs669*: NeuroPAL | This study |
| OH17012 | *nlp-42(syb3238[nlp-42::T2A::3xNLS::GFP]); otIs669; him-5(e1490) V* |  | This study |
| OH17068 | *tab-1(ok2198) II; nlp-42(syb3238); otIs669 him-5(e1490) V* |  | This study |
| OH17037 | *otIs576; otIs669; him-5(e1490) V; ttx-3(ot22) X* |  | This study |
| OH17053 | *otIs669 him-5(e1490) V; ttx-3(ot22) X; nlp-42 (syb3238)* |  | This study |
| NY2050 | *ynIs50 (pflp-22::GFP); him-5(e1490)* |  | [32] |
| OH17973 | *ynIs50 (pflp-22::GFP); ceh-14(ot900)* |  | [32] |
| PHX4413 | *flp-27(syb4413 [flp-27::SL2::GFP::H2B])* |  | This study |
| PHX3212 | *flp-21(syb3212 [flp-21::T2A::3×NLS::GFP])* |  | This study |
| PHX3411 | *nlp-13(syb3411 [nlp-13::T2A::3XNLS::GFP])* |  | This study |
| OH17974 | *flp-27(syb4413 [flp-27::SL2::GFP::H2B]); ceh-14(ot900)* |  | This study |
| OH17975 | *flp-21(syb3212 [flp-21::T2A::3×NLS::GFP]); ceh-14(ot900)* |  | This study |
| OH17976 | *nlp-13 (syb3411 [nlp-13::T2A::3XNLS::GFP]); ceh-14(ot900)* |  | This study |
| PHX5421 | *ins-3(syb5421[ins-3::SL2::gfp::H2B]) II.* |  | This study |
| PHX5463 | *ins-6(syb5463[ins-6::SL2::gfp::H2B]) II.* |  | This study |
| PHX5447 | *ins-24(syb5447[ins-24::SL2::gfp::H2B]) I* |  | This study |
| PHX5526 | *ins-30(syb5526[ins-30::SL2::gfp::H2B]) I* |  | This study |
| PHX5697 | *nlp-2(syb5697 [nlp-2::SL2::GFP::H2B]) X* |  | This study |
| NY2067 | *ynIs67 III; him-5(e1490) V.* | *flp-6prom::GFP* | [32] |
| NY2037 | *ynIs37* | *flp-13prom::GFP* | [32] |
| OH17795 | *ceh-36(gj2127) X* |  | [20] |
| RB823 | *ceh-37(ok642) X* |  | [33] |
| PHX3936 | *nlp-51(syb3936[nlp-51::SL2::GFP::H2B])* |  | This study |
| PHX4406 | *nlp-73(syb4406[nlp-73::SL2::GFP::H2B])* |  | This study |
| OH17968 | *nlp-51(syb3936[nlp-51::SL2::GFP::H2B]); unc-86(ot1158)* |  | This study |
| OH17992 | *unc-86(ot1158); nlp-73(syb4406[nlp-73::SL2::GFP::H2B])* |  | This study |
| OH17957 | *nlp-51(syb3936[nlp-51::SL2::GFP::H2B]); unc-86(ot1158); ttx-1(p767)* |  | This study |
| OH16366 | *otEx7603* | *nlp-52p::gfp, pha-1(+)* | This study |
| OH18015 | *nlp-51(syb3936[nlp-51::SL2::GFP::H2B])*; ceh-14(ot900) |  | This study |
| OH18016 | *nlp-73(syb4406[nlp-73::SL2::GFP::H2B])*; ceh-14(ot900) |  | This study |
| OH18075 | *nlp-51(syb3936[nlp-51::SL2::GFP::H2B]); mls-2(cc615)* |  | This study |
| OH18076 | *nlp-73(syb4406[nlp-73::SL2::GFP::H2B]); mls-2(cc615)* |  | This study |
| OH16866 | *otIs810* | *otIs810* *[sto-3promRIB::tagRFP;*  *sto-3promRIB::GFP:cla-1(S)]* | This study |
| OH17016 | *otEx7705* | *otEx7705[unc-17p9::NLS:RFP]* | This study; [13] |
| OH18119 | *vab-3(ot1269) X; otEx7705* |  | This study |
| OH18111 | *ttx-1(syb1679[ttx-1::GFP] ot1264)* |  | This study |
| OH18112 | *ttx-1(ot1265); nlp-73(syb4406[nlp-73::SL2::GFP::H2B])* |  | This study |
| OH18113 | *ttx-1(ot1266); unc-86(ot1158); nlp-73(syb4406[nlp-73::SL2::GFP::H2B])* |  | This study |
| OH18115 | *ttx-1(ot1267); unc-86(ot1158); nlp-51(syb3997[nlp-51::SL2::GFP::H2B])* |  | This study |
| OH18122 | *egl-5(ot1271); kyIs37* |  | This study |

**References for strain list**

1. Ma X, Zhao Z, Xiao L, Xu W, Kou Y, Zhang Y, et al. A 4D single-cell protein atlas of transcription factors delineates spatiotemporal patterning during embryogenesis. Nat Methods. 2021;18(8):893-902. Epub 2021/07/28. doi: 10.1038/s41592-021-01216-1. PubMed PMID: 34312566.

2. Furuya M, Qadota H, Chisholm AD, Sugimoto A. The C. elegans eyes absent ortholog EYA-1 is required for tissue differentiation and plays partially redundant roles with PAX-6. Dev Biol. 2005;286(2):452-63. Epub 2005/09/13. doi: 10.1016/j.ydbio.2005.08.011. PubMed PMID: 16154558.

3. Brenner S. The genetics of Caenorhabditis elegans. Genetics. 1974;77(1):71-94.

4. Chalfie M, Au M. Genetic control of differentiation of the Caenorhabditis elegans touch receptor neurons. Science. 1989;243(4894 Pt 1):1027-33. PubMed PMID: 2646709.

5. Jiang Y, Horner V, Liu J. The HMX homeodomain protein MLS-2 regulates cleavage orientation, cell proliferation and cell fate specification in the C. elegans postembryonic mesoderm. Development. 2005;132(18):4119-30. Epub 2005/08/19. doi: 10.1242/dev.01967. PubMed PMID: 16107479.

6. Vidal B, Gulez B, Cao WX, Leyva-Diaz E, Reilly MB, Tekieli T, et al. The enteric nervous system of the C. elegans pharynx is specified by the Sine oculis-like homeobox gene ceh-34. eLife. 2022;11. Epub 2022/03/25. doi: 10.7554/eLife.76003. PubMed PMID: 35324425; PubMed Central PMCID: PMCPMC8989417.

7. Serrano-Saiz E, Poole Richard J, Felton T, Zhang F, De La Cruz Estanisla D, Hobert O. Modular Control of Glutamatergic Neuronal Identity in C. elegans by Distinct Homeodomain Proteins. Cell. 2013;155(3):659-73. doi: <https://doi.org/10.1016/j.cell.2013.09.052>.

8. Sengupta P, Chou JH, Bargmann CI. odr-10 encodes a seven transmembrane domain olfactory receptor required for responses to the odorant diacetyl. Cell. 1996;84(6):899-909. Epub 1996/03/22. doi: S0092-8674(00)81068-5 [pii]. PubMed PMID: 8601313.

9. McIntire SL, Reimer RJ, Schuske K, Edwards RH, Jorgensen EM. Identification and characterization of the vesicular GABA transporter. Nature. 1997;389(6653):870-6.

10. Reilly MB, Cros C, Varol E, Yemini E, Hobert O. Unique homeobox codes delineate all the neuron classes of C. elegans. Nature. 2020;584(7822):595-601. Epub 2020/08/21. doi: 10.1038/s41586-020-2618-9. PubMed PMID: 32814896.

11. Tsalik EL, Niacaris T, Wenick AS, Pau K, Avery L, Hobert O. LIM homeobox gene-dependent expression of biogenic amine receptors in restricted regions of the C. elegans nervous system. Dev Biol. 2003;263(1):81-102. PubMed PMID: 14568548.

12. Tobin D, Madsen D, Kahn-Kirby A, Peckol E, Moulder G, Barstead R, et al. Combinatorial expression of TRPV channel proteins defines their sensory functions and subcellular localization in C. elegans neurons. Neuron. 2002;35(2):307-18. Epub 2002/08/06. doi: S0896627302007572 [pii]. PubMed PMID: 12160748.

13. Serrano-Saiz E, Gulez B, Pereira L, Gendrel M, Kerk SY, Vidal B, et al. Modular Organization of Cis-regulatory Control Information of Neurotransmitter Pathway Genes in Caenorhabditis elegans. Genetics. 2020;215(3):665-81. Epub 2020/05/24. doi: 10.1534/genetics.120.303206. PubMed PMID: 32444379; PubMed Central PMCID: PMCPMC7337081.

14. Leyva-Diaz E, Hobert O. Robust regulatory architecture of pan-neuronal gene expression. Curr Biol. 2022;32:1715–27. Epub 2022/03/09. doi: 10.1016/j.cub.2022.02.040. PubMed PMID: 35259341.

15. Taylor SR, Santpere G, Weinreb A, Barrett A, Reilly MB, Xu C, et al. Molecular topography of an entire nervous system. Cell. 2021;184(16):4329-47 e23. Epub 2021/07/09. doi: 10.1016/j.cell.2021.06.023. PubMed PMID: 34237253.

16. Cros C, Hobert O. Caenorhabditis elegans sine oculis/SIX-type homeobox genes act as homeotic switches to define neuronal subtype identities. Proc Natl Acad Sci U S A. 2022;119(37):e2206817119. Epub 2022/09/07. doi: 10.1073/pnas.2206817119. PubMed PMID: 36067313; PubMed Central PMCID: PMCPMC9478639.

17. Gendrel M, Atlas EG, Hobert O. A cellular and regulatory map of the GABAergic nervous system of C. elegans. eLife. 2016;5. doi: 10.7554/eLife.17686. PubMed PMID: 27740909; PubMed Central PMCID: PMC5065314.

18. Yemini E, Lin A, Nejatbakhsh A, Varol E, Sun R, Mena GE, et al. NeuroPAL: A Multicolor Atlas for Whole-Brain Neuronal Identification in C. elegans. Cell. 2021;184(1):272-88 e11. Epub 2020/12/31. doi: 10.1016/j.cell.2020.12.012. PubMed PMID: 33378642.

19. Finney M. The Genetics and Molecular Biology of unc-86, a C.elegans cell lineage gene [Ph.D.]. Cambridge, MA: Massachusetts Institute of Technology; 1987.

20. Traets JJ, van der Burght SN, Rademakers S, Jansen G, van Zon JS. Mechanism of life-long maintenance of neuron identity despite molecular fluctuations. eLife. 2021;10. Epub 2021/12/16. doi: 10.7554/eLife.66955. PubMed PMID: 34908528; PubMed Central PMCID: PMCPMC8735970.

21. Lloret-Fernandez C, Maicas M, Mora-Martinez C, Artacho A, Jimeno-Martin A, Chirivella L, et al. A transcription factor collective defines the HSN serotonergic neuron regulatory landscape. eLife. 2018;7. doi: 10.7554/eLife.32785. PubMed PMID: 29553368.

22. Flames N, Hobert O. Gene regulatory logic of dopamine neuron differentiation. Nature. 2009;458(7240):885-9. Epub 2009/03/17. doi: nature07929 [pii] 10.1038/nature07929. PubMed PMID: 19287374.

23. Doitsidou M, Flames N, Topalidou I, Abe N, Felton T, Remesal L, et al. A combinatorial regulatory signature controls terminal differentiation of the dopaminergic nervous system in C. elegans. Genes Dev. 2013;27(12):1391-405. doi: 27/12/1391 [pii] 10.1101/gad.217224.113. PubMed PMID: 23788625.

24. Schwartz HT, Horvitz HR. The C. elegans protein CEH-30 protects male-specific neurons from apoptosis independently of the Bcl-2 homolog CED-9. Genes Dev. 2007;21(23):3181-94. doi: 10.1101/gad.1607007. PubMed PMID: 18056428; PubMed Central PMCID: PMC2081982.

25. Nass R, Hall DH, Miller DM, 3rd, Blakely RD. Neurotoxin-induced degeneration of dopamine neurons in Caenorhabditis elegans. Proc Natl Acad Sci U S A. 2002;99(5):3264-9. PubMed PMID: 11867711.

26. Tekieli T, Yemini E, Nejatbakhsh A, Varol E, Fernandez R, Masoudi N, et al. Visualizing the organization and differentiation of the male-specific nervous system of C. elegans. BioRxiv. 2021.

27. Bayer E, Hobert O. A novel null allele of C. elegans gene ceh-14. MicroPubl Biol. 2018;2018. Epub 2018/10/18. doi: 10.17912/g434-3d85. PubMed PMID: 32550390; PubMed Central PMCID: PMCPMC7282514.

28. Gordon PM, Hobert O. A Competition Mechanism for a Homeotic Neuron Identity Transformation in C. elegans. Dev Cell. 2015;34(2):206-19. doi: 10.1016/j.devcel.2015.04.023. PubMed PMID: 26096732; PubMed Central PMCID: PMC4519388.

29. Gray JM, Hill JJ, Bargmann CI. A circuit for navigation in Caenorhabditis elegans. Proc Natl Acad Sci U S A. 2005;102(9):3184-91. Epub 2005/02/04. doi: 0409009101 [pii]

10.1073/pnas.0409009101. PubMed PMID: 15689400; PubMed Central PMCID: PMC546636.

30. Sarov M, Murray JI, Schanze K, Pozniakovski A, Niu W, Angermann K, et al. A genome-scale resource for in vivo tag-based protein function exploration in C. elegans. Cell. 2012;150(4):855-66. doi: 10.1016/j.cell.2012.08.001. PubMed PMID: 22901814; PubMed Central PMCID: PMC3979301.

31. Brunschwig K, Wittmann C, Schnabel R, Burglin TR, Tobler H, Muller F. Anterior organization of the Caenorhabditis elegans embryo by the labial-like Hox gene ceh-13. Development. 1999;126(7):1537-46. PubMed PMID: 10068646.

32. Kim K, Li C. Expression and regulation of an FMRFamide-related neuropeptide gene family in Caenorhabditis elegans. The Journal of comparative neurology. 2004;475(4):540-50. doi: 10.1002/cne.20189. PubMed PMID: 15236235.

33. Consortium CeDM. large-scale screening for targeted knockouts in the Caenorhabditis elegans genome. G3 (Bethesda). 2012;2(11):1415-25. Epub 2012/11/23. doi: 10.1534/g3.112.003830. PubMed PMID: 23173093; PubMed Central PMCID: PMCPMC3484672.
